# Transcriptome profiling of aging *Drosophila* photoreceptors reveals gene expression trends that correlate with visual senescence

**DOI:** 10.1186/s12864-017-4304-3

**Published:** 2017-11-21

**Authors:** Hana Hall, Patrick Medina, Daphne A. Cooper, Spencer E. Escobedo, Jeremiah Rounds, Kaelan J. Brennan, Christopher Vincent, Pedro Miura, Rebecca Doerge, Vikki M. Weake

**Affiliations:** 1 Department of Biochemistry, Purdue University, West Lafayette, IN 47907 USA; 2 Department of Statistics, Purdue University, West Lafayette, IN 47907 USA; 3 Department of Biology, University of Nevada, Reno, NV 89557 USA; 4 Carnegie Mellon University, Pittsburgh, PA 15213 USA; 5 Purdue University Center for Cancer Research, Purdue University, West Lafayette, 47907 USA

**Keywords:** Aging, Transcriptome, Drosophila, Neurons, Photoreceptors

## Abstract

**Background:**

Aging is associated with functional decline of neurons and increased incidence of both neurodegenerative and ocular disease. Photoreceptor neurons in *Drosophila melanogaster* provide a powerful model for studying the molecular changes involved in functional senescence of neurons since decreased visual behavior precedes retinal degeneration. Here, we sought to identify gene expression changes and the genomic features of differentially regulated genes in photoreceptors that contribute to visual senescence.

**Results:**

To identify gene expression changes that could lead to visual senescence, we characterized the aging transcriptome of *Drosophila* sensory neurons highly enriched for photoreceptors. We profiled the nuclear transcriptome of genetically-labeled photoreceptors over a 40 day time course and identified increased expression of genes involved in stress and DNA damage response, and decreased expression of genes required for neuronal function. We further show that combinations of promoter motifs robustly identify age-regulated genes, suggesting that transcription factors are important in driving expression changes in aging photoreceptors. However, long, highly expressed and heavily spliced genes are also more likely to be downregulated with age, indicating that other mechanisms could contribute to expression changes at these genes. Lastly, we identify that circular RNAs (circRNAs) strongly increase during aging in photoreceptors.

**Conclusions:**

Overall, we identified changes in gene expression in aging *Drosophila* photoreceptors that could account for visual senescence. Further, we show that genomic features predict these age-related changes, suggesting potential mechanisms that could be targeted to slow the rate of age-associated visual decline.

**Electronic supplementary material:**

The online version of this article (10.1186/s12864-017-4304-3) contains supplementary material, which is available to authorized users.

## Background

The incidence of ocular disease increases with age leading to an increase in reported visual impairment from 5.7% in 18 – 44 year old people to 21% in people older than 75 years [1]. Whereas theories of aging in the eye have traditionally focused on the role of oxidative damage to the genome and mitochondrial dysfunction [2], changes in expression of genes in the aging retina could also contribute to the age-associated increase in disease susceptibility [3]. Photoreceptor neurons, and in particular rod photoreceptors, which comprise the major retinal cell type in humans, show age-associated decreases in both visual function and in number [4–14]. Loss of rod photoreceptors is the major factor leading to ocular disease-associated blindness [15]. Microarray analysis of aging mouse rod photoreceptors indicates that gene expression changes begin as early as five months of age in rodents, preceding pathological changes by two years [12, 13]. Thus, gene expression changes precede the onset of visual dysfunction and disease. Identifying these signature early gene expression changes could therefore provide the opportunity to prevent or delay the onset of ocular disease.

As in humans, the fruitfly *Drosophila melanogaster* shows visual senescence, defined as a progressive decline in visual function with age. While young flies are attracted towards light and show positive phototaxis, this phototactic behavior decreases with age [16–18]. Moreover, the rate of visual senescence is influenced by genetic variation [16], indicating that genetic factors regulate the age-related loss of visual function. Here, we sought to identify gene expression changes and the genomic features of differentially regulated genes in aging photoreceptor neurons that contribute to visual senescence.

Since the retina is comprised of multiple cell types, we focused our analysis on the outer photoreceptor neurons in *Drosophila*, R1 – R6 cells. These six outer photoreceptor neurons share several key functional similarities with human rods. First, both cell types represent the majority of photoreceptors in the retina, function in dim light, and express a single rhodopsin protein, Rhodopsin 1 (Rh1) [19]. Second, phototransduction in both human rods and in R1 – R6 photoreceptor cells initiates with the light-induced isomerization of photosensitive rhodopsin [20]. The rapid life cycle of *Drosophila*, coupled with our ability to genetically label and isolate photoreceptors in an intact organism, permitted us to examine the photoreceptor transcriptome at multiple time points during aging, prior to the first signs of retinal degeneration. Here, we show that subsets of genes in photoreceptor neurons are age-regulated. We find that combinations of sequence motifs and gene characteristics such as gene length and exon content identify age-regulated genes. Further, we show that circular RNAs (circRNAs) accumulate in aged photoreceptors. Together, these data indicate that targeting gene expression mechanisms might provide a way to slow the rate of visual decline associated with aging and thereby delay the onset of ocular disease.

## Results

### Visual function declines with age independent of retinal degeneration

**Fig. 1:**
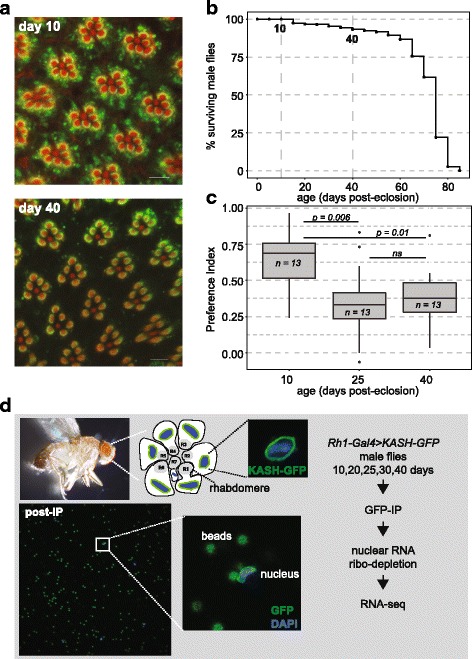
Visual function declines with age independent of retinal degeneration. **a** Representative confocal images of adult retinas stained with phalloidin (red) and 4C5 (Rh1, green) from male *Rh1-Gal4 > KASH-GFP* flies 10 and 40 days post-eclosion (*n* = 5). Scale bars: 5 μm. **b** Survival curve showing the percentage of viable *Rh1-Gal4 > KASH-GFP* male flies at each age (*n* = 345). **c** Box plots showing the light preference indices (positive phototaxis) for *Rh1-Gal4 > KASH-GFP* flies at day 10, 25 and 40 (*n* = 13 experiments; 27 - 33 male flies/experiment). *p* value, normally-distributed data were analyzed using ANOVA followed by Tukey’s honest significant different (HSD) post hoc test. **d** Photoreceptor R1 – R6 nuclei in each ommatidium were labeled with nuclear membrane-localized GFP in *Rh1-Gal4 > KASH-GFP* flies. Affinity-enriched GFP-labeled nuclei bound to antibody-coated magnetic beads are shown in the two lower panels. DAPI, blue; GFP, green. Schematic of the RNA-seq experimental design is shown in the right panel. Graphic generated by authors

In this study, we sought to identify gene expression changes in aging photoreceptor neurons that could contribute to visual senescence. While decreased visual behavior is observed by 3 – 4 weeks of age, little retinal degeneration is observed in wild-type flies at these ages [16, 17, 21, 22]. We directly compared retinal degeneration, phototaxis and lifespan in *Rh1-Gal4 > KASH-GFP* flies to identify an age at which flies show decreased visual behavior in the absence of either retinal degeneration or significant morbidity. Male flies were used for all experiments because sex-specific differences have been reported for both phototaxis and visual senescence [16, 17]. Similar to observations from other groups [22], we found that 99.6% and 99.2% of rhabdomeres in the outer photoreceptors (R1 - R6) are intact in male flies at day 10 and day 40 post-eclosion (emergence from the pupal case), respectively (*n* = 5; Fig. 1a). Rhabdomere loss is observed during retinal degeneration in flies, and provides a stringent measure of photoreceptor health; thus, we conclude that there is no significant retinal degeneration by 40 days post-eclosion. In addition, 93% of male flies survived until day 40 under our standard laboratory conditions with 12:12 h light/dark cycles (Fig. 1b). While we did not observe significant morbidity or retinal degeneration by day 40, we did observe a significant decrease in positive phototaxis in male day 40 flies compared to day 10 flies using a two-choice T-maze assay (Fig. 1c). Significantly decreased phototaxis was also observed in day 25 flies relative to day 10; however, day 25 flies also showed more variability in phototaxis as compared with either the day 10 or day 40 flies, suggesting that these flies are more heterogeneous with respect to visual behavior. Although older flies are known to have decreased locomotion [17], the T-maze assay minimizes the effect of locomotive behavior on phototaxis since flies are presented with a single choice between light and dark [23]. In addition, published reports show that the age-related increase in visual senescence reflects visual behavior rather than locomotion [16]. Thus, day 40 flies show decreased visual behavior in the absence of retinal degeneration, indicating that cellular function is compromised in the aging eye.

### Transcriptome profiling of photoreceptor neurons

The *Drosophila* eye consists of repeating units termed ommatidia that each contain about 20 different cells including eight photoreceptor neurons [24, 25]. We sought to profile the transcriptome of aging photoreceptors to identify genes that show age-dependent changes in expression in this visual cell type. Photoreceptors are highly polarized epithelial cells that extend axons to the neuropils in the brain [24]. To profile the photoreceptor transcriptome, we examined nuclear RNA, which has been shown to correlate well with levels of active transcription [26]. To do this, we labeled photoreceptor nuclei with nuclear membrane-localized GFP (KASH-GFP). Whereas the outer photoreceptors (R1 – R6) express Rh1, the inner photoreceptors (R7 and R8) express Rh3/4 and Rh5/6 respectively [19, 27]. We labeled R1 – R6 photoreceptors using *Rh1-Gal4* [28] driven *UAS-KASH-GFP*. The Klarsicht, Anc-1, Syn3-1 homology (KASH) domain of Msp300 localizes GFP to the cytoplasmic face of the nuclear membrane, allowing subsequent affinity-enrichment of labeled nuclei with GFP antibodies coupled to magnetic beads (Fig. 1d) [29, 30].

To determine the enrichment of our target nuclei versus nonspecific background levels, we mixed equal numbers of flies that expressed either *KASH-GFP* or *KASH-mCherry* in photoreceptors under *Rh1-Gal4* control, and generated head homogenates in which an equal number of GFP- and mCherry-labeled nuclei were present. We then performed GFP affinity-enrichment, and measured GFP and mCherry transcript levels in the pre-isolation (head homogenate) and post-isolation samples by qPCR. We observed 82 ± 22 fold enrichment of GFP transcripts in the post-isolation samples, with no corresponding increase in mCherry levels, demonstrating that the affinity-enrichment of GFP-labeled nuclei is highly specific (Additional file 1: Figure S1A). Next, we profiled the transcriptome of affinity-enriched GFP-labeled nuclei from 10 day old male flies using Illumina sequencing (RNA-seq) and compared this to the corresponding pre-isolation samples. The post-isolation samples for each of the three biological replicates grouped together by principal component analysis and were distinct from each of their respective pre-isolation samples (Additional file 1: Figure S1B). Using edgeR, we identified 447 post-enriched and 444 post-reduced genes (False Discovery Rate, FDR < 0.05, fold change, FC > 2) (Additional file 1: Figure S1C, Additional file 2: Table S1). Supporting an enrichment of photoreceptors, gene ontology (GO) term analysis of the 447 photoreceptor post-enriched genes identified terms including phototransduction, calcium signaling and retina homeostasis (Additional file 1: Figure S1D, Additional file 3: Table S2). In contrast, the post-reduced genes were enriched for a variety of metabolic pathways. Consistent with a depletion of cytoplasmic and mitochondrial RNAs in affinity-enriched nuclear RNA, 11 of 13 detected mitochondrial-encoded genes were significantly reduced in the post-isolation samples. These data demonstrate that RNA isolated using our approach is highly enriched for nuclear RNA transcribed in the target cell population.

Surprisingly, we identified the GO term *Sensory perception of sound* (GO:0007605) as being significantly overrepresented in our post-enriched gene group. This GO term shares a number of common gene members with the GO terms describing phototransduction and light response including the R7 – R8 rhodopsins *Rh5* and *Rh6* (Additional file 1: Figure S1D). The auditory organ (Johnston’s organ: JO) in *Drosophila* is composed of a sound receiver and an auditory sensory organ that contains mechanosensory neurons [31]. Intriguingly, several genes that function in phototransduction are expressed in the auditory organ including *Arr2, Rh3, Rh5, Rh6, inaD, trp* and *trpl* [32]. Since, *Rh1* transcription is restricted to R1 – R6 photoreceptors, which do not express other rhodopsins [27, 33–37], we hypothesized that expression of *Rh1-Gal4* in mechanosensory neurons could account for the observed enrichment of R7 – R8 rhodopsins in our study. To determine if *Rh1-Gal4* was expressed in antennae, we purified total RNA from dissected heads, eyes, antennae and bodies of *Rh1-Gal4 > KASH-GFP* flies and examined *Rh1* and *GFP* transcript levels by qPCR. In line with our hypothesis, we found that both *Rh1* and *GFP* genes are expressed in the antennae at ~10 - 20% levels found in the eye (Additional file 1: Figure S2). This therefore accounts for enrichment of R7 – R8 photoreceptor-specific markers such as *Rh3* and *Rh5* in our affinity-enriched photoreceptor nuclei. We note that similar enrichment of R7 – R8 *rhodopsins* was previously reported by Yang et al. who profiled R1 – R6 mRNAs by expressing polyA-binding protein under *Rh1-Gal4* control and purifying bound-mRNAs from whole heads [38]. Since there are approximately 9600 outer photoreceptor neurons and 1000 mechanosensory neurons per head [25, 39], we conclude that using *Rh1-Gal4 > KASH-GFP* flies, our approach predominantly enriches photoreceptor neurons, but that ~10% of our enriched nuclei are most likely contributed by mechanosensory neurons.

### Age-related changes in the photoreceptor transcriptome

To identify genes that show age-regulated expression in photoreceptors, we affinity-enriched *Rh1-Gal4 > KASH-GFP* labeled nuclei from adult male flies. To avoid changes in gene expression associated with the transition from development to adulthood, and to identify changes in gene expression that contribute to decreased phototaxis between day 10 and 40, we profiled the photoreceptor nuclear transcriptome at 10, 20, 25, 30 and 40 days post-eclosion (Fig. 1d). We obtained similar RNA yields across each time point (Additional file 1: Figure S3A) that yielded an average of 32 million high-quality paired-reads for each biological replicate (*n* = 3). We discarded one sample (day 30 replicate 3) due to poor alignment. We then analyzed the RNA-seq time series data using maSigPro, which is a generalized linear model-based approach [40]. Utilizing maSigPro and multiple time points enabled us to identify genes with robust expression changes that correlate strongly with chronological age. Notably, maSigPro has a much lower false positive rate for time series data when compared with pair-wise differential expression methods such as edgeR [41]. Using maSigPro, we identified 604 age-regulated genes (FDR < 0.05). To limit the age-regulated genes to those that were expressed specifically in photoreceptors, we excluded 49 age-regulated genes that were significantly reduced in the post-isolation samples from day 10 flies (Additional file 2: Table S1). Thus, 555 genes were differentially expressed with age in *Drosophila* photoreceptors (Additional file 4: Table S3). This differential expression did not reflect differences in the relative GFP-labeling of photoreceptors and mechanosensory neurons because GFP mRNA and protein levels in the eye did not change with age (Additional file 1: Figure S3B,C). Moreover, we did not observe consistent patterns of change in expression of neuronal cell-type specific genes during aging (Additional file 1: Figure S3D). Further, 5 of 7 selected age-regulated genes showed significant differences in expression between day 10 and 40 in dissected eyes from male flies and in independent affinity-enriched samples by qPCR (Additional file 1: Figure S4). Thus, the majority of age-regulated genes identified are differentially expressed in photoreceptors. Phototaxis differs between male and female flies with one study reporting 20% lower phototaxis in females at 4 weeks, while another showed 10% higher phototaxis at the same age [16, 21]. Further, a recent study has shown that age-related changes in gene expression in the retina differ between male and female mice [42]. To test if female flies showed similar patterns of gene expression changes in aging photoreceptors, we examined a subset of the age-regulated genes by qPCR in female eyes. We observed the same trends in gene expression for the age-regulated genes examined between day 10 and 40 in dissected eyes from male and female flies (Additional file 1: Figure S4). However, these gene expression changes were not significant in the female flies due both to high variability in the young samples, and to smaller magnitude of changes between young and old eyes. Thus, we conclude that gene expression changes in aging photoreceptors are likely similar between male and female flies, but that female flies might show delayed onset of these gene expression changes compared with males.

**Fig. 2:**
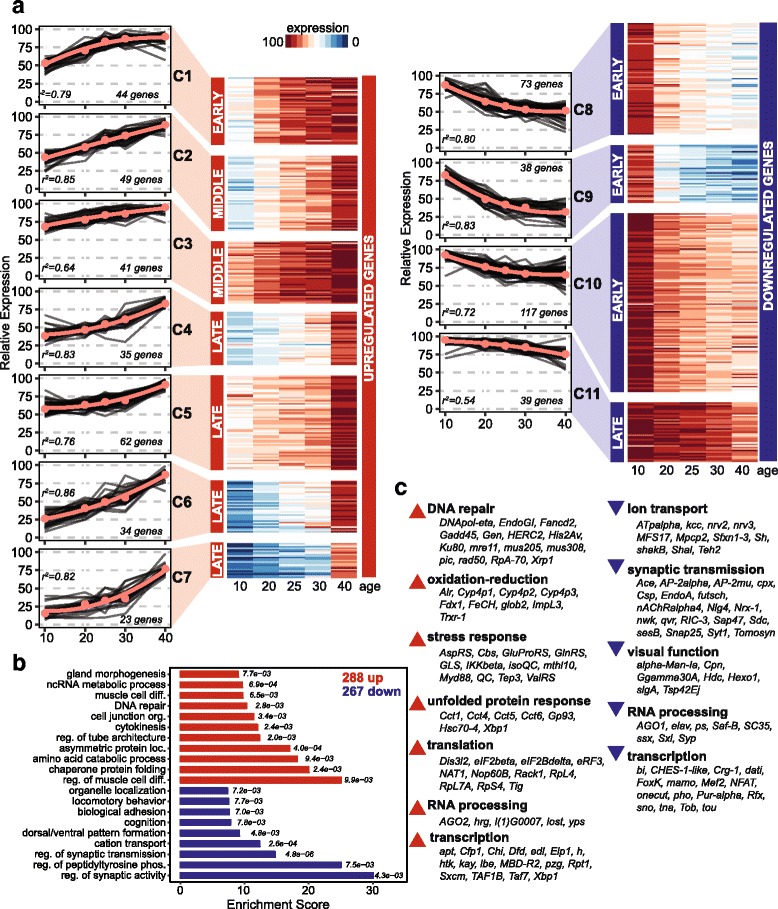
Age-related changes in gene expression in adult photoreceptors. **a** Age-regulated genes identified by time-series analysis using maSigPro (555 genes, FDR < 0.05) were clustered using k-means into 11 clusters based on temporal expression pattern (relative expression). The median expression values (circles) and fitted curves with indicated r^2^ values are shown in red on the line graphs. Age-regulated genes were designated as upregulated or downregulated, and early, middle or late based on the fitted curves for their respective cluster (see Additional file 1: Fig. S4). **b** Over-represented GO terms (*p* < 0.01, Fisher’s exact test) were identified for 288 upregulated or 267 downregulated genes relative to all 7579 expressed genes using TopGO (Additional file 5: Table S4). Similar GO terms were grouped based on intersecting gene members, and a single representative GO term is shown from each group in the bar plot. Enrichment score indicates the number of genes with the GO term in the target gene set versus the number of expected genes, with *p*-values shown to the right of each bar. **c** Representative functional categories identified using GO term analysis and DAVID for upregulated and downregulated genes. Selected age-regulated genes involved in the indicated functions are shown below each term based on published reports

We next characterized the direction and temporal pattern of the changes in gene expression for the age-regulated genes. To do this, we used k-means clustering to group the 555 age-regulated genes based on their temporal pattern of gene expression. We found that the age-regulated genes clustered into 11 expression clusters, whose changes in relative gene expression over age were best described by second degree polynomial equations (Fig. 2a). Only moderate improvement was obtained from using higher order polynomials or by increasing the number of clusters used for k-means clustering (Additional file 1: Figure S5A). We next determined the overall direction of the change in gene expression for age-regulated genes in each clusters based on the slope of the fitted curve: using these criteria, 288 age-regulated genes were upregulated, and 267 age-regulated genes were downregulated by day 40 (Additional file 1: Figure S5B). We then determined when the change in gene expression occurred: early clusters showed maximal changes in expression between days 10 and 20, late clusters between days 30 and 40, while the middle clusters showed little to no change in the rate of expression (slope) throughout the time course. Most of the downregulated genes were found in the early clusters, with only 39 genes (cluster 11) being downregulated late. In contrast, only 44 of the upregulated genes fell into an early cluster, while 154 upregulated genes were in late clusters. Thus, the temporal expression clustering suggests that most age-related changes in gene expression in photoreceptors do not occur gradually or linearly. Instead, most downregulated genes showed the highest rates of changes in gene expression at the earliest stages of the aging process, while more than half of upregulated genes increased later during aging. These data suggest that distinct mechanisms underlie the changes in gene expression observed in these subsets of age-regulated genes.

### Aging is associated with upregulation of stress-inducible genes and downregulation of genes required for neuronal function

Next, we asked if the gene expression changes observed in aging photoreceptors could contribute to the observed visual senescence between day 10 and days 25 and 40. GO term analysis of the upregulated genes revealed an enrichment for genes indicative of an induced stress response such as DNA repair and the unfolded protein response (Fig. 2b, Additional file 5: Table S4). In contrast to the upregulated genes, the downregulated genes were enriched for GO terms including ion transport, synaptic transmission and behavior (Fig. 2b, Additional file 5: Table S4). Further analysis using DAVID [43] identified additional functional categories associated with the age-regulated genes; we then examined published reports to identify specific age-regulated genes involved in these processes which could potentially impact visual function (Fig. 2c).

In-depth analysis of the age-regulated genes revealed that multiple genes in the DNA damage response pathway were upregulated with age including those that function in non-homologous end-joining repair (*mre11*, *rad50*, *Ku80* and *mus308*) and in translesion DNA synthesis (*mus205* and *DNApol-eta*) [44–46]. Genes that encoded enzymes with antioxidant properties, such as the thioredoxin reductase *Trxr-1*, and antioxidant genes involved in glutamate metabolism, such as *GlnRS*, *isoQC* and *QC*, were also upregulated with age [47–50]. We also observed increased age-associated expression of chaperone genes (*Cct1*, *Cct4*, *Cct5*, *Cct6*, *Hsc70-4*) and the unfolded protein response transcription factor *Xbp1*, consistent with an induction of the unfolded protein response [51–53]. Under stress conditions, there is a translational switch that favors production of stress-related proteins while decreasing translation of other proteins [54]. Paralogs of canonical translation factors such as *NAT1* and *Rack1*, which were both upregulated, promote this switch to cap-independent translation [55, 56]. Notably, *Rheb*, which is downregulated with age, positively regulates ribosome production and cap-dependent translation by activating the mechanistic target of rapamycin (mTOR) kinase pathway [57]. Thus, decreased *Rheb* levels during aging could decrease mTOR pathway activity, which extends lifespan and is protective against age-related pathology [58]. Together, these data suggest that multiple genes are induced in aging photoreceptors to mitigate the effects of oxidative stress, protein misfolding and DNA damage.

In contrast to the upregulated genes, many of the genes that were downregulated with age are required for the proper response to light in photoreceptors. For example, sodium and potassium ion channels, such as those encoded by *Atpα, Sh, shakB* and *Shal,* are required for the sensitivity and dynamic range of photoreceptors in response to bright light [59–62]. In addition, ion channels, such as Sh, and genes such as *Csp, Hdc* and *Sap47* are necessary for proper synaptic function, which is required to transmit the light signal from the retina to the brain [63–66]. Further, calcium-binding proteins such as Cpn function in photoreceptors to buffer potentially toxic levels of intracellular calcium induced by prolonged phototransduction [67–70]. Several other genes including *Hexo1*, *α-Man-Ia* and *Tsp42Ej* encode proteins required for post-translational modification or degradation of Rh1 [71, 72]. Notably, mutations that prevent either processing of Rh1, or degradation of activated endocytosed Rh1, cause retinal degeneration [72–75]. In addition to genes that are necessary for photoreceptor function, some of the age downregulated genes have been directly shown to impact phototaxis including *Ace*, *slgA* and *Rheb* [76–78]. Together, these data show that the cumulative downregulation of genes involved in processes required for photoreceptor function could indeed account for the decrease in vision we observed by days 25 and 40.

Intriguingly, functional analysis using DAVID showed an enrichment for RNA processing and transcription in both the up- and downregulated genes. Several splicing factor genes including *ps*, Saf*-B* and *SC35* were downregulated with age [79, 80]. In contrast, *hrg*, which encodes the single poly(A) polymerase enzyme required for mRNA polyadenylation in flies [81], is upregulated with age. Further, whereas *AGO1*, which regulates microRNA-induced silencing is downregulated with age, *AGO2*, which regulates small interfering-RNA silencing is upregulated [82]. *AGO2* has been linked to repair of double-stranded DNA breaks [83], suggesting that changes in expression of some RNA processing factors might occur as part of the stress response. In addition to genes involved in RNA processing, a large number of genes that encode transcription factors or transcriptional regulatory proteins were age regulated. Some of these transcription factors could control expression of other age-regulated genes. For example, the calcium-regulated transcription factor NFAT, which is downregulated with age, is required for neural development, including pre-synaptic growth, and plasticity [84]. Similarly, the transcription factor onecut that is required to maintain neuronal identity, is also downregulated with age [85]. Thus, decreases in levels of these transcription factors could contribute to downregulation of neuronal-specific genes, such as those involved in synaptic transmission. Similarly, upregulation of *Xbp1* could contribute to upregulation of genes involved in the UPR, although Xbp1 activity is primarily regulated through alternative splicing [86]. In addition to transcription factors, epigenetic regulators such as the TFIID subunit Taf7 or the Set1/COMPASS histone methyltransferase subunit Cfp1 are also age regulated [87, 88]. These data suggest that multiple factors converge to drive changes in the transcriptional landscape of aging photoreceptors.

### Combinations of promoter sequence motifs identify age-regulated genes

We sought to identify factors involved in the regulation of gene expression that drive changes in the transcriptional landscape of aging photoreceptors. Since several transcription factors are themselves age-regulated, and because alterations in age-related signaling pathways converge on transcription factors, we first asked whether the age-regulated genes were targeted by common transcription factors. To do this, we examined the upregulated genes (clusters 1 – 7) or the downregulated genes (clusters 8 – 11) to identify shared promoter sequence motifs. We used HOMER (Hypergeometric Optimization of Motif EnRichment) [89] to identify significantly enriched (*p* < 0.001) sequence motifs in the promoters of age up- or downregulated genes. Using this approach, 40 significantly enriched sequence motifs were identified for the upregulated genes and 41 significantly enriched motifs were identified for the downregulated genes (Additional file 6: Table S5).

**Fig. 3:**
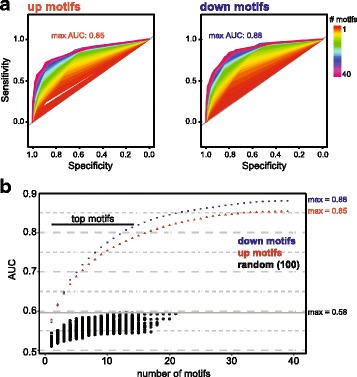
Combinations of promoter motifs identify age-regulated genes. **a** Receiver operating characteristic (ROC) curves for combinations of promoter motifs that identify age-regulated genes. Significantly-enriched promoter sequence motifs for up- or downregulated genes were identified using HOMER (40 motifs upregulated genes, 41 motifs downregulated genes; Additional file 6: Table S5). ROC curves representing the diagnostic ability of each motif to identify whether a gene would be up- or downregulated were compared, and the motif with the highest area under the curve (AUC) was iteratively combined with other motifs to identify motif combinations. The maximum AUC values obtained for combinations of motifs are shown. **b** The AUC values for ROC curves generated by combining increasing numbers of motifs for up- or downregulated genes as described in panel A. The addition of a single motif does not significantly improve the ROC curve (*p* < 0.05) after the first 14 motifs; we define the first 14 motifs as the top motifs. The maximum AUC value obtained for ROC curves based on 40 randomly-assigned motifs was 0.58 (100 random iterations, see methods)

We then asked if the presence of any combination of the individual enriched sequence motifs were associated with a gene that is up or downregulated with age. To do this, we generated ROC (Receiver Operating Characteristic) curves to assess the ability of each individual sequence motif to identify whether a gene would be up or downregulated with age. We then compared the AUC (area under the curve) for each ROC curve; higher AUC values indicate an improved ability to identify genes that were age-regulated. Not surprisingly, no single motif provided a strong ability to identify the direction of regulation with age. However, when we analyzed combinations of promoter motifs generated by iteratively combining the motif with the highest AUC score with other motifs, we found that increasing combinations of sequence motifs could strongly identify whether a gene would be up or downregulated with age (Fig. 3a). A combination of all 40 sequence motifs resulted in AUC values of 0.88 or 0.85 for the up and downregulated genes respectively, versus 0.58 for the control (see methods); the AUC value for the motif combination was significantly improved by the addition of a single motif for the first 14 motifs, which we termed *top motifs* (Fig. 3b, Additional file 1: Figure S6). Network analysis indicated that these top motifs co-occurred frequently with other motifs at gene promoters (Additional file 1: Figure S7A). Further, the top motifs showed lower clustering coefficients in the network, indicating that they co-occurred with a wider variety of the other enriched-motifs (Additional file 1: Figure S7B). Together, these data are consistent with combinatorial activity of transcription factors at age-regulated genes, and suggest that the top motifs identified represent binding sites for transcription factors that integrate multiple types of signaling pathways during aging.

Next, we asked which transcription factors were most likely to bind the top sequence motifs. To do this, we used HOMER to compare the top motifs with known insect transcription factor binding sites including those recently identified by Nitta et al. [90]. We then asked whether the putative transcription factor matches were expressed in photoreceptors based on our RNA-seq data. A complete list of matching, expressed transcription factors is provided for all top motifs in Additional file 7: Table S6, and the best matches for the top motifs based on score are described in Additional file 1: Figure S6. The age-regulated genes with promoter motifs putatively bound by these transcription factors are shown in Additional file 1: Figure S8 and Figure S9. We observed differences in the occurrence of specific motifs in each expression cluster, suggesting that different groups of transcription factors regulate early and late genes (Additional file 1: Figure S10). Further, several of the transcription factors that match the top motifs were themselves age regulated at the gene expression level. For example, the transcription repressor hairy (*h*) [91] was upregulated early during aging, and matched one of the top motifs (motif 15) identified for the downregulated genes. In contrast, the transcription activator onecut [85], which was downregulated early during aging, matched one of the top motifs (motif 9) for the downregulated genes. The transcription factor Deformed (*Dfd*) was present in one of the middle upregulated gene clusters (cluster 2), and matched one of the top motifs for the upregulated genes (motif 17). Another early upregulated gene, the FOS homolog *kayak* (kay) that together with Jun-related antigen (*Jra*) forms the AP-1 transcription factor [92], corresponded to another of the top motifs for the upregulated genes (motif 18). AP-1 acts downstream of Jun-N-terminal kinase signaling in response to UV-induced DNA damage in the *Drosophila* retina [93], consistent with a putative role for AP-1 in upregulating stress-responsive aging genes such as the DNA repair gene *Xrp1* (Additional file 1: Figure S9). Together, these data suggest that transcription factors play a key role in driving the gene expression changes observed in aging photoreceptors.

### Gene length, expression and splicing correlate with age-downregulation

Several data suggest that the regulation of transcription elongation and RNA processing events could also contribute to age-related gene expression changes. Neuronal genes tend to be longer than average [94], and are often heavily alternatively spliced [95], implying that transcription elongation or splicing might be critical for proper expression of these genes. Expression of long genes is more dependent on topoisomerases, which relieve transcription-induced torsional stress [96], and on proper DNA repair because long genes stochastically accumulate more DNA damage [97]. Our data indicate that genes involved in splicing are age-regulated in photoreceptors. Notably, age-related changes in splicing, which could contribute to alterations in mRNA levels, have been observed in several studies [98]. Since the genes that are downregulated during aging include a large number of neuron-specific genes, we wondered whether a bias in their genomic features, such as gene length or number of exons, could contribute to their age-associated decline.

To examine this question, we analyzed gene length, overall expression level across all time points (RPKM), and the number of expressed exons and transcript isoforms for genes in each expression cluster C1 – C11 (Additional file 1: Figure S11). We used Wilcoxon Rank-Sum test to compare pair-wise differences in the distribution of gene length or other characteristics between each expression cluster and the genes that were not age-regulated (non-significant genes). Three of the 4 downregulated clusters showed significantly different distributions of gene length, expression or transcription isoform number compared with the nonsignificant genes. In each of these downregulated clusters, genes showed longer median gene lengths, higher median expression, and higher median numbers of expressed transcript isoforms. Further, 2 of the 4 downregulated clusters had significantly different distributions of exon numbers, with higher median numbers of expressed exons. In contrast, only one of the upregulated clusters showed significantly different distribution of expression (higher median expression) or transcript isoforms (smaller median number). Although increased expression level and gene length could contribute to enhanced statistical power in differential gene expression analysis [99], we would expect this statistical power to apply equally to genes in all of the expression clusters whether they were up or downregulated. Thus, these data demonstrate that the age down-regulated genes show a bias towards long, highly expressed and heavily spliced genes.

**Fig. 4:**
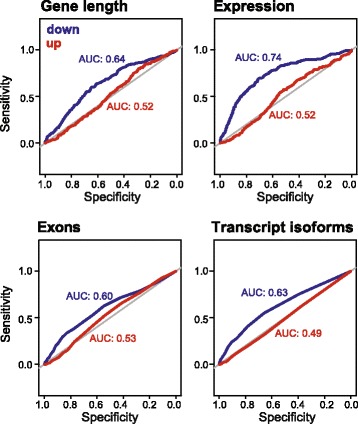
Gene length, expression and splicing correlate with age-related downregulation. ROC curves for gene length including introns, expression (RPKM), number of expressed exons and transcripts isoforms for down or upregulated genes. AUC values are indicated for each curve

Next, we asked if any of the characteristics that showed a bias in the downregulated gene set could identify whether a given gene would be downregulated with age. To do this, we generated ROC curves based on the ability of each characteristic, such as gene length, to identify whether a gene would be up- or downregulated (Fig. 4). Whereas the ROC curves for gene length, expression, exon number or transcript isoforms showed no ability to identify upregulated genes, all four characteristics showed modest ability to identify downregulated genes with AUCs between 0.60 – 0.74. These data suggest that long, highly transcribed genes are susceptible to downregulation with age, implying that in addition to transcription factors, other gene regulatory mechanisms might become altered with age.

### circRNA levels strongly correlate with age in photoreceptors

**Fig. 5:**
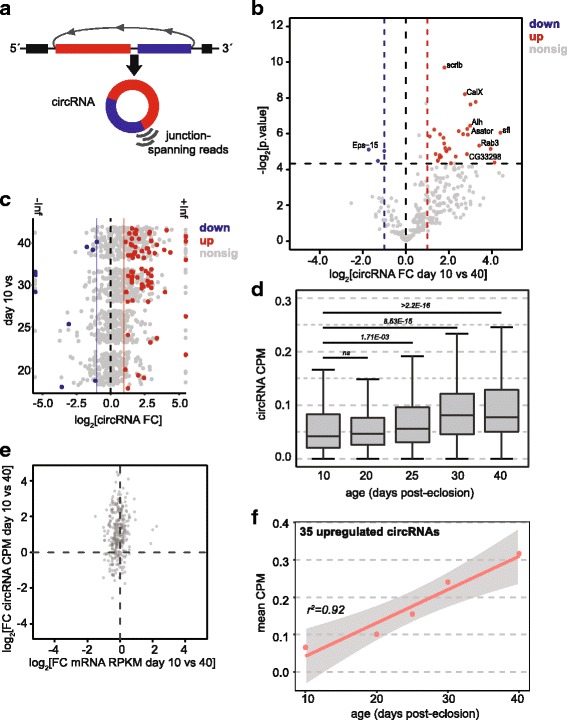
circRNA levels increase in aging photoreceptors. **a** Schematic showing how junction-spanning reads were used to detect circRNAs resulting from back-splicing events. **b** Volcano plot showing the fold change in circRNA abundance plotted as log_2_(fold change in counts per million reads, CPM) for each circRNA relative to its *p* value (−log_2_[p.value]). CircRNAs with significantly differential expression (*p* ≤ 0.05 and FC ≥ 2, dotted lines) are highlighted. Labels indicate the corresponding host gene for selected circRNAs. **c** Fold changes in circRNA abundance for pairwise comparisons between the indicated aging time-point. CircRNAs with significantly differential expression (*p* ≤ 0.05 and FC ≥ 2, blue/red) are highlighted. **d** Total abundance of circRNAs (CPM) identified at each age. *p* values, non-parametrical Kruskal-Wallis with Nemenyi post-hoc test for multiple comparisons. **e** Density plots comparing the log_2_ fold changes in circRNA CPM with fold change in linear RNA RPKM from the corresponding gene for 10 versus 40 day sensory neurons. **f** Linear regression analysis of the mean CPM of the 35 significantly upregulated circRNAs versus age

In addition to changes in the expression of specific genes during aging, specific classes of RNA known as circRNAs show increased abundance with age [100]. These circRNAs are generated from back-splicing events at known splicing sites of protein coding genes, lack free 5′ and 3′ ends, and are highly stable because they cannot be degraded by cellular exoribonucleases [101] (Fig. 5a). While circRNA abundance has been shown to increase between day 1 and day 20 in *Drosophila* heads [100], it has not been demonstrated whether circRNA abundance continues to increase linearly with age. Further, although circRNA accumulation in neurons is thought to underlie the age-associated increase in abundance, whether this accumulation can occur in the nucleus has not been demonstrated. We sought to examine whether circRNA abundance would increase linearly with age due to chronological accumulation of circRNAs in photoreceptor neurons. To do this, we used CIRI2 to identify circRNAs from our affinity-enriched photoreceptor transcriptome data [102, 103]. We identified 1209 circRNAs in the sensory neuron data with at least two counts from the 14 libraries (Additional file 8: Table S7). For these circRNAs, 1095 were previously annotated [100], and 114 were novel annotations. We quantified circRNA abundance as counts per million reads (CPM), and calculated pairwise differential expression statistics across all pair-wise comparisons. Using this approach, we identified 38 out of 315 abundant circRNAs (>6 total counts per circRNA) that were differentially expressed between day 10 and 40 (*p* < 0.05) (Fig. 5b, Additional file 8: Table S7). Thirty-five out of these 38 circRNAs increased with age, supporting an overall increase in circRNA abundance with age. Notably, there was a strong trend for circRNA accumulation that was not statistically significant most likely due to low read counts (Fig. 5b, bottom right quadrant). When we examined levels of a subset of the identified age-regulated circRNAs in independent samples from day 10 and day 40 flies, we validated that 6/6 circRNAs that were significantly increased in the RNA-seq data also showed significant increases in expression by RT-qPCR (Additional file 1: Figure S12). The single downregulated circRNA examined, *Eps-15*, did not show significantly decreased expression by RT-qPCR. We conclude that the downregulated circRNAs identified are likely false positives, and that our analysis probably underestimates the number of circRNAs that are upregulated with age (also see [104]).

Next, we performed pairwise comparisons to identify significantly age-regulated circRNAs between the different ages. Notably, a comparison between day 10 and day 30 or 40 identified many more significantly upregulated circRNAs than day 10 compared with day 20 or 25 (Fig. 5c). Next, we plotted expression of all detected circRNAs that met a minimum read cutoff of one unique read per library (14 read count minimum per circRNA) for all time points, and compared circRNA abundance between day 10 and each subsequent time point (Fig. 5d). Wilcoxon rank sum test with continuity correction revealed significant increases in circRNA abundance during aging between days 10 and 25, 30 or 40, but not between day 10 and 20. Importantly, changes in levels of individual circRNAs between days 10 and 40 occurred independent of the host gene mRNA (Fig. 5e), indicating that changes in expression of the host gene do not influence circRNA levels. Thus, we conclude that either enhanced biogenesis through increased back-splicing, or exceptional stability of circRNAs underlies their age-dependent accumulation. While we cannot distinguish between these possibilities, the genes from which abundant circRNAs are generated were significantly longer and more heavily spliced than all other genes (Additional file 1: Figure S13), suggesting that any alterations in splicing would be likely to affect circRNA biogenesis. Although circRNA host genes shared these characteristics (gene length, splicing) with the age downregulated genes, only 27 of the 218 genes with highly-expressed circRNA were significantly downregulated with age, whereas 5 circRNA host genes were upregulated. Together, these data suggest that circRNA biogenesis does not substantially influence expression of most host genes.

If circRNA abundance progressively increases with age, then we would expect that overall circRNA levels would correlate with age in photoreceptor neurons. Indeed, the mean circRNA expression level (CPM) for the 35 age upregulated circRNAs correlated highly with age (Fig. 5f, r^2^ = 0.92). Because the photoreceptor transcriptome data is based on nuclear RNA, and showed lower levels of cytoplasmic transcripts such as mitochondrial genes, the increase in circRNA abundance with age is likely to reflect circRNAs that are retained in the nucleus. Together, these results suggest that the increased circRNA levels observed in aging heads may be driven by accumulation of circRNAs in neurons, including photoreceptors.

## Discussion

In this study, we describe gene expression changes in aging photoreceptor neurons that correlate with visual senescence. Many genes required for neuronal function, such as those involved in ion transport or synaptic transmission, are downregulated in photoreceptors during aging. Moreover, the decreased expression of these genes begins at the earliest stages of the aging process when decreased phototaxis is first observed (day 25), and precedes the upregulation of most stress-response genes. The age-related decrease in expression of genes required for neuron function is evolutionarily conserved across a variety of organisms including flies, worms and vertebrates [105]; the mouse retina shows decreased expression of phototransduction genes with age [106, 107], and there is reduced expression of genes involved in synaptic plasticity in the brain of elderly humans [108–110]. Since 40 day old flies show no significant retinal degeneration, the decreased expression of genes required for neuronal function could account for the decreased phototaxis observed in these flies. Humans and rhesus macaques show much stronger age-dependent repression of neuronal genes in the brain than mice, leading to the conclusion that repression mechanisms have evolved recently [109]. However, genes involved in synaptic transmission are downregulated in our aging photoreceptor data, and in two independent studies from aging *Drosophila* heads [111, 112]. Similar to observations from aging gene expression studies in other tissues [98], we observed a general upregulation of stress response genes, including unfolded protein response and DNA damage response genes in aging photoreceptors. Notably, these changes occurred mostly mid- to late in aging photoreceptors, subsequent to the downregulation of many of the neuronal-specific genes discussed above.

Interestingly, 380 of the 555 age-regulated genes in *Drosophila* photoreceptors have identifiable human homologs, six of which are associated with retinal disease (RetNet: http://www.sph.uth.tmc.edu/RetNet/): *Cyp4c3* (*CYP4V2*), *l(1)G0007* (*DHX38*), *CG5291* (*PDZD7*), *CG3662* (*ITM2B*), *krz* (*SAG*), and *Cct1* (*PCYT1A*). For example, the human homolog of the age down-regulated gene *Cyp4c3*, *CYP4V2*, is associated with a recessive inherited retinal disorder, Bietti crystalline corneoretinal dystrophy that involves progressive age-associated retinal dystrophy. In addition, heterozygous mutation in *ITM2B*, the human homolog of the age down-regulated gene *CG3662*, is associated with another late-onset retinal dystrophy. The human homolog of *l(1)G0007*, *DHX38* (*PRP16*), which encodes an ATP-dependent RNA helicase involved in splicing that is upregulated with age, is associated with a recessive early-onset form of retinitis pigmentosa. The age-regulated genes with human homologs associated with retinal disease provide additional candidate genes that could impact either visual function or photoreceptor survival at older ages.

Here, we show that combinations of promoter motifs strongly predict whether a gene will be up- or downregulated with age, indicating an important role of transcription factors in driving changes in the transcriptional landscape of aging photoreceptors. We find that three of the transcription factors that provide best matches to the top motifs for the downregulated genes are annotated as being negative regulators of transcription (*Negative regulation of transcription from RNA polymerase II promoter, GO:0000122; rn, h, ovo*) whereas seven transcription factors are described as positive regulators of transcription (*Positive regulation of transcription from RNA polymerase II promoter, GO:0045944; sd, Cf2, br, ovo, onecut, SoxN, Adf1*). Similarly, three transcription factors that match the top motifs for the upregulated genes are negative regulators (*Dfd, dsx, Blimp-1*), whereas eight are positive regulators (*Dfd, Mad, kay, br, dsx, Trl, vvl, Mef2*). These data suggest that alterations in both transcription activation and repression are involved in the gene expression changes observed in aging photoreceptors. We note that caution should be used in definitively assigning specific transcription factors to each of the top motifs, since some of the lower scoring transcription factor matches for particular motifs might be more biologically relevant to aging; matches assigned in this study represent highest scoring matches for expressed transcription factors. For example, the CHES-1-like transcription factor is a close match to top motifs for both the upregulated (motif 8) and downregulated (motif 19) genes, but is outscored by broad (*br*) in both instances (Additional file 7: Table S6). CHES-1-like is required for hypoxia-induced inhibition of protein translation in cultured *Drosophila* cells [113], whereas broad is expressed in neurons and is required for some behaviors in *Drosophila* [114, 115]. Thus, both CHES-1-like and broad represent candidate transcription factors that could bind top motifs in the up or downregulated genes, and functional studies would be required to determine whether either of these transcription factors bound the target genes that we identified via motif analysis in photoreceptors. An additional limitation of the motif analysis used in this study was that we restricted our search to sequence motifs within 500 bp of the transcription start site. Many transcription factors are known to have longer range effects, and our sequence motif analysis would not identify these factors. Gene network analysis of the age-regulated genes using GeneMania reveals some additional transcription factors and signaling kinases that could be involved in the mechanisms regulating these genes (Additional file 9: Table S8). For example, the transcription factor *crooked legs* (*crol*) and the MAP kinase *misshapen* (*msn*) are not age-regulated at the transcript level in photoreceptors, but are highly co-regulated with the age-regulated genes in terms of physical and genetic interactions, and co-expression analysis.

One of the challenges in identifying mechanisms that could drive age-related changes in gene expression is the cellular heterogeneity present in many aging gene expression studies [98]. Previous studies of gene expression in *Drosophila* heads using microarrays compared ages ranging from day 1 to 80 [111, 116, 117]; recently, age-regulated genes were also identified in heads using RNA-seq [112, 118]. A comparison of our data with the 2914 age-regulated genes identified in the most recent RNA-seq study showed an overlap of only 348 of our 555 age-regulated genes [112]. These data suggest that our cell-type specific approach identifies a subset of age-regulated genes that are masked by the cellular heterogeneity present in whole heads. However, the differences in the age-regulated genes identified might also reflect the inherent difficulty in comparing the nuclear and cytoplasmic transcriptomes. Since the nuclear enrichment strategy used in our approach biases the data towards genes that are actively transcribed, age-associated changes in the storage pool of cytoplasmic mRNAs available for translation are not reflected in our current photoreceptor data. However, we note that our qPCR analysis of individual age-regulated genes in dissected eyes largely mirrored the results from photoreceptor nuclei. Complementary cell type-specific data on the ribosome-bound pool of mRNAs destined for translation could be obtained using the translating ribosome affinity purification (TRAP) technique, based on cell-specific expression of an EGFP-tagged ribosomal subunit [119, 120].

## Conclusion

The long-lived, post-mitotic nature of neurons makes them uniquely vulnerable to the long-term accumulation of genomic damage and oxidative stress. Here, we show that gene expression changes in photoreceptors precede retinal degeneration and correlate with observed decreases in visual function. Further, we demonstrate that the transcriptional landscape of aging photoreceptors is driven in large part by transcription factors. Notably, highly expressed, long and heavily spliced genes show a bias towards downregulation while circRNA levels increase strongly with age, suggesting that other gene expression mechanisms become altered with age. The identification of potential regulatory mechanisms that drive changes in photoreceptors provides targets to delay gene expression changes in aging neurons, and thereby postpone the onset of ocular disease.

## Methods

### Fly strains, aging and phototaxis assays

Flies homozygous for KASH-GFP, *P{w* ^*+mC*^ *= UAS-GFP-Msp300KASH}attP2*, under the control of Rh1-Gal4 (*P{ry* ^*+t7.2*^ *= rh1-GAL4}3, ry* ^*506*^, BL8691] were raised in 12:12 h light:dark cycle at 25 °C on standard fly food [121]. For aging studies, flies were collected on the day of eclosion (day 1) and transferred to fresh vials every two days. For RNA-seq studies, 400 male flies were harvested between 10 am and 12 pm on the indicated day for each time point. For the survival curve, male flies were counted every 5 days until death. For phototaxis assays, 27-33 male flies were tested per assay (*n = 13 assays*) using a custom-built T-maze apparatus [23, 122] with dark equilibration time of 10 min and choice time of 2 min.

### Immunostaining and western blotting

Retinal degeneration was assessed by confocal microscopy of adult retinas immunostained with phalloidin (Thermo Fisher; cat# A22287) and anti-Rhodopsin 1 (1:50, Developmental Studies Hybridoma Bank, cat# 4C5). Detailed protocols are provided via PURR. Western blotting analysis was performed using 40 μg of protein extracted from dissected eyes using the following antibodies: anti-GFP (rabbit; BioVision; 1:1000; cat# 3992).

### Nuclei immuno-enrichment

For each sample, 400 male adult flies were anesthetized, frozen in liquid nitrogen and stored at −80 °C. For nuclei isolation, frozen flies were submitted to five rounds of vortexing and cooling in liquid nitrogen and heads were separated from thoracicoabdominal segments, wings and legs using two different-sized pre-chilled sieves (Hogentogler, 710 μm and 425 μm pore sizes). Separated heads were transferred into 1 mL of Nuclei Extraction Buffer (15 mM Hepes [Na+], pH 7.5, 10 mM KCl, 5 mM MgCl_2_) in a Dounce homogenizer and incubated on ice for 5 min. Nuclei were extracted by using five strokes with a loose pestle, followed by an incubation on ice for 5 min and subsequent 5 strokes with a loose pestle. Head homogenate was filtered through a 40 μm Falcon cell strainer (VWR, cat # 21008-949) and immunoprecipitated with 10 μg of GFP antibody (Roche, cat # 11814460001) as previously described [29] with the following modifications: The salt concentration in the PBS wash buffer was supplemented to a final concentration of 300 mM NaCl, and five washes were conducted. Detailed protocols are provided via PURR.

### RNA isolation and qPCR analysis

RNA for RNA-seq experiments was isolated using Trizol (Invitrogen). Quantitative real time PCR (qPCR) analysis for mRNA or circRNAs were performed on independent post-IP samples for day 10 and 40 relative to a standard curve of serially diluted cDNA generated using random hexamers as previously described [29]. qPCR analysis of heads, eyes, antennae and bodies from mixed flies was performed on total RNA isolated using Direct-zol RNA Micro-prep kit (Zymo Research, Cat. # R2062). Relative expression for each gene was normalized to the geometric mean of two reference genes based on MIQE guidelines [123]. Primers are listed in Additional file 10: Table S9.

### Transcriptome library construction and high throughput sequencing (RNA-seq)

The cDNA libraries were generated from 10 ng of total nuclear RNA using the NuGEN Ovation RNA seq Systems 1-16 for Model Organism (NuGEN, cat #0350). RNA was DNAse treated, and single-stranded DNA was generated using both random hexamer and oligo-dT primers, and then depleted for ribosomal DNA. The cDNA libraries were ligated to unique adaptors and multiplexed libraries were sequenced with Illumina HiSeq 2500 technology. Three biological replicates were analyzed for each sample. Day 30 sample #3 was subsequently discarded due to poor mapping. Single-end 50 bp reads were sequenced for the post and pre day 10 samples, and paired-end 100 bp reads were sequenced for all aging samples (days 10 – 40).

### RNA-seq data analysis

Reads were trimmed using Trimmomatic (v0.36). Quality trimmed reads were mapped to the *D. melanogaster* genome (BDGP6.89) using bowtie-2 (v2.3.2) and Tophat (v2.1.1). Counts were identified for each gene or exon using Htseq-count (v0.7.1) with strand-specific conditions (fr-secondstrand) and default parameters. Differential expression analysis was performed on genes with CPM > 1 in at least three of the samples. Differentially expressed genes between post and pre samples were identified using the *glmTreat* function in edgeR (v3.18.1) [124] with a FDR < 0.05 and FC > 2. Age-regulated genes were identified using maSigPro [40] with a FDR < 0.05 and clustered using k-means clustering based on relative mean expression values (maximum normalized count value set to one) for each time point.

### Functional annotation analysis

All functional enrichment analyses were performed relative to the background gene set of all expressed genes with CPM > 1 in at least three of the samples. GO term enrichment analysis on post-enriched or reduced genes was performed using topGO (v2.28.0) [125]. Related significantly-enriched GO terms were grouped using hierarchical clustering based on shared gene members. The DAVID functional annotation tool [43] was used to identify enriched functional classes in the age-regulated genes using the default parameters. Human homologs of *Drosophila* age-regulated genes were identified using the RetNet Database (RetNet, http://www.sph.uth.tmc.edu/RetNet/).

### Motif analysis

Significantly-enriched promoter motifs were identified separately for up and downregulated genes using HOMER (v4.9, Hypergeometric Optimization of Motif EnRichment) [89] with the following parameters: motif length of 8, 10 or 12 bp within 500 bp upstream or downstream from the transcription start site. Motif enrichment analyses were performed relative to the background gene set of all expressed genes with CPM > 1 in at least three of the samples (7580 genes). The presence (1) or absence (0) of each of the 40 (up) or 41 (down) significantly-enriched motifs identified was determined for each gene in the background gene set, and these data were used for subsequent ROC analysis using pROC (v1.10.0) [126]. As a control, the maximum AUC computed from 100 random matrices was used. Each of the matrices were composed of 7580 rows (genes) with either presence (1) or absence (0) of a motif assigned based on random probabilities between 0 and 0.145 (the maximum number of genes in the background gene set matching to any specific motif was 14.5%). Sequence motifs were plotted using seqLogo (v1.42.0). Network analysis for co-occurring motifs was performed using igraph (v1.0.1), and for age-regulated genes using GeneMania [127]. To identify candidate transcription factors that bind each sequence motif, we compared the top motifs to the HOMER insect transcription factor database using the default parameters. The position weight matrices for an additional recently characterized 242 *Drosophila* transcription factors were added to the HOMER insect database [90].

### Gene characteristic analysis

The total gene length (including introns) was determined as the gene length of the most abundant expressed transcript isoform. Transcript abundance and exon expression (exons with count >1 in at least one sample) were determined using exon counts obtained using Htseq-count from the photoreceptors aging RNA-seq data. Expression levels (RPKM) represent mean RPKM value across all time points and samples for a given gene. Kruskal–Wallis tests were performed to identify significant differences in the distribution of gene characteristics between any group. Pairwise Wilcoxon Rank Sum Tests were then performed to identify which groups exhibited significant differences in the distribution of the examined characteristic and FDR values were determined using a Benjamini and Hochberg correction. ROC analysis was performed using pROC (v1.10.0) [126].

### circRNA analysis

Trimmed reads were also used as input for CIRI2 [102, 103] to identify circRNAs that mapped to annotated splice sites. A circRNA junction scaffold of 170 nts in length (85 nts of the downstream exonic junction and 85 nts of the upstream exonic junction) was generated for each circRNA using Bedtools getfasta [128]. Reads were mapped to the circRNA junction scaffold with Bowtie2 using the following option *--score-min = C,-15,0*. PCR and sequencing duplicates were removed using Picard MarkDuplicates (http://broadinstitute.github.io/picard/). Custom scripts were used to ensure that reads overlapped the circRNA junction by a minimum of 15 nts. Reads were assigned to individual circRNA records using Featurecounts [129], and only circRNA records with a minimum average of 1 read per library were used for analysis (i.e. 6 read minimum across 6 libraries). Read counts were normalized to CPM to account for library size variation. For pairwise comparisons between time points, we required a minimum of one count per library (i.e. six read minimum cut-off between day 10 and 40).

### Database accession numbers

RNA-seq expression data are available in the Gene Expression Omnibus (GEO) repository through GEO series accession numbers GSE93128 and GSE83431. Raw data, detailed protocols and R custom scripts used for analysis have been deposited in the Purdue University Research Repository (PURR) as a publically available, archived data set and can be accessed using 10.4231/R7736P29.

## Additional files

Additional file 1: Complete Supplemental Figures. **Figure S1.** Affinity-purified nuclear RNA is enriched for photoreceptor-expressed genes. **Figure S2.** Rh1-Gal4 drives GFP expression in antennal sensory neurons. **Figure S3.** Relative sensory neuron proportions and yields of affinity-purified nuclear RNA do not change with age. **Figure S4.** qPCR of selected age-regulated genes. **Figure S5.** K-means clustering of age-regulated genes based on temporal expression pattern. **Figure S6.** Top promoter motifs that predict age-related expression changes. **Figure S7.** Promoter motifs with the best predictive power co-occur frequently with a variety of other motifs. **Figure S8.** Distribution of the top motifs in age upregulated genes. **Figure S9.** Distribution of the top motifs in age upregulated genes. **Figure S10.** Distribution of the top motifs between expression clusters. **Figure S11.** Downregulated gene clusters are enriched for longer, more highly expressed and more heavily spliced genes. **Figure S12.** qPCR of selected age-regulated circRNAs. **Figure S13.** circRNA-containing host genes are enriched for longer and more heavily spliced genes. (PDF 4807 kb)

Additional file 2: Table S1. Significantly post-enriched or post-reduced genes in affinity-enriched photoreceptor nuclear RNA. (XLSX 89 kb)

Additional file 3: Table S2. GO term analysis of significantly post-reduced genes. (XLSX 16 kb)

Additional file 4: Table S3. Age-regulated genes in photoreceptor neurons. (XLSX 121 kb)

Additional file 5: Table S4. GO term analysis of age-regulated genes in photoreceptor neurons. (XLSX 14 kb)

Additional file 6: Table S5. Enriched sequence motifs for age-regulated genes. (XLSX 48 kb)

Additional file 7: Table S6. Transcription factors matches for top motifs identified for age-regulated genes. (XLSX 13 kb)

Additional file 8: Table S7. circRNA lists. (XLSX 96 kb)

Additional file 9: Table S8. GeneMania analysis of age-regulated genes in photoreceptors. (XLSX 174 kb)

Additional file 10: Table S9. Primers used in this study. (XLSX 13 kb)

## Abbreviation

AUC: Area under the curve
circRNA: Circular RNA
CPM: Counts per million
GEO: Gene Expression Omnibus
GFP: Green fluorescent protein
GO: Gene ontology
HOMER: Hypergeometric Optimization of Motif EnRichment
HSD: Honest significant different
KASH: Klarsicht, Anc-1, Syn3-1 homology
mTOR: Mechanistic target of rapamycin
qPCR: Quantitative PCR
Rh1: Rhodopsin 1
ROC: Receiver Operating Characteristic
RPKM: Reads per kilobase per million reads
TRAP: Translating ribosome affinity purification

## References

[1] Klein R, Klein BE (2013). The prevalence of age-related eye diseases and visual impairment in aging: current estimates. Invest Ophthalmol Vis Sci.

[2] Alavi MV (2016). Aging and vision. Adv Exp Med Biol.

[3] Yang HJ, Ratnapriya R, Cogliati T, Kim JW, Swaroop A (2015). Vision from next generation sequencing: multi-dimensional genome-wide analysis for producing gene regulatory networks underlying retinal development, aging and disease. Prog Retin Eye Res.

[4] Curcio CA, Millican CL, Allen KA, Kalina RE (1993). Aging of the human photoreceptor mosaic: evidence for selective vulnerability of rods in central retina. Invest Ophthalmol Vis Sci.

[5] Curcio CA (2001). Photoreceptor topography in ageing and age-related maculopathy. Eye (Lond).

[6] Gao H, Hollyfield JG (1992). Aging of the human retina. Differential loss of neurons and retinal pigment epithelial cells. Invest Ophthalmol Vis Sci.

[7] Birch DG, Anderson JL (1992). Standardized full-field electroretinography. Normal values and their variation with age. Arch Ophthalmol.

[8] Bonnel S, Mohand-Said S, Sahel JA (2003). The aging of the retina. Exp Gerontol.

[9] Freund PR, Watson J, Gilmour GS, Gaillard F, Sauve Y (2011). Differential changes in retina function with normal aging in humans. Doc Ophthalmol.

[10] Shinomori K, Werner JS (2012). Aging of human short-wave cone pathways. Proc Natl Acad Sci U S A.

[11] Gresh J, Goletz PW, Crouch RK, Rohrer B (2003). Structure-function analysis of rods and cones in juvenile, adult, and aged C57bl/6 and Balb/c mice. Vis Neurosci.

[12] Kolesnikov AV, Fan J, Crouch RK, Kefalov VJ (2010). Age-related deterioration of rod vision in mice. J Neurosci.

[13] Parapuram SK, Cojocaru RI, Chang JR, Khanna R, Brooks M, Othman M, Zareparsi S, Khan NW, Gotoh N, Cogliati T (2010). Distinct signature of altered homeostasis in aging rod photoreceptors: implications for retinal diseases. PLoS One.

[14] Samuel MA, Zhang Y, Meister M, Sanes JR (2011). Age-related alterations in neurons of the mouse retina. J Neurosci.

[15] Wright AF, Chakarova CF, Abd El-Aziz MM, Bhattacharya SS (2010). Photoreceptor degeneration: genetic and mechanistic dissection of a complex trait. Nat Rev Genet.

[16] Carbone MA, Yamamoto A, Huang W, Lyman RA, Meadors TB, Yamamoto R, Anholt RR, Mackay TF (2016). Genetic architecture of natural variation in visual senescence in drosophila. Proc Natl Acad Sci U S A.

[17] Simon AF, Liang DT, Krantz DE (2006). Differential decline in behavioral performance of Drosophila Melanogaster with age. Mech Ageing Dev.

[18] Grotewiel MS, Martin I, Bhandari P, Cook-Wiens E (2005). Functional senescence in Drosophila Melanogaster. Ageing Res Rev.

[19] Rister J, Desplan C (2011). The retinal mosaics of opsin expression in invertebrates and vertebrates. Dev Neurobiol.

[20] Montell C (2012). Drosophila visual transduction. Trends Neurosci.

[21] Lebourg E, Badia J (1995). Decline in photopositive tendencies with age in drosophila-Melanogaster (Diptera, Drosophilidae). J Insect Behav.

[22] Kurada P, O'Tousa JE (1995). Retinal degeneration caused by dominant rhodopsin mutations in drosophila. Neuron.

[23] Gorostiza EA, Colomb J, Brembs B. A decision underlies phototaxis in an insectE. Axel Gorostiza, Julien Colomb, Björn Brembs Open Biol. 2016;6:160229. doi:10.1098/rsob.160229. Published 21 December 2016 10.1098/rsob.160229 PMC5204122 28003472

[24] Katz B, Minke B (2009). Drosophila photoreceptors and signaling mechanisms. Front Cell Neurosci.

[25] Ready DF, Hanson TE, Benzer S (1976). Development of the Drosophila Retina, a neurocrystalline lattice. Dev Biol.

[26] Steiner FA, Talbert PB, Kasinathan S, Deal RB, Henikoff S (2012). Cell-type-specific nuclei purification from whole animals for genome-wide expression and chromatin profiling. Genome Res.

[27] Chou WH, Hall KJ, Wilson DB, Wideman CL, Townson SM, Chadwell LV, Britt SG (1996). Identification of a novel drosophila opsin reveals specific patterning of the R7 and R8 photoreceptor cells. Neuron.

[28] Yoshihara Y, Mizuno T, Nakahira M, Kawasaki M, Watanabe Y, Kagamiyama H, Jishage K, Ueda O, Suzuki H, Tabuchi K (1999). A genetic approach to visualization of multisynaptic neural pathways using plant lectin transgene. Neuron.

[29] Ma J, Weake VM. Affinity-based isolation of tagged nuclei from drosophila tissues for gene expression analysis. J Vis Exp. 2014;85 10.3791/51418 PMC4158730 24686501

[30] Ma J, Brennan KJ, D'Aloia MR, Pascuzzi PE, Weake VM (2016). Transcriptome profiling identifies Multiplexin as a target of SAGA Deubiquitinase activity in Glia required for precise axon guidance during drosophila visual development. G3 (Bethesda).

[31] Gopfert MC, Robert D (2001). Biomechanics. Turning the key on drosophila audition. Nature.

[32] Senthilan PR, Piepenbrock D, Ovezmyradov G, Nadrowski B, Bechstedt S, Pauls S, Winkler M, Mobius W, Howard J, Gopfert MC (2012). Drosophila auditory organ genes and genetic hearing defects. Cell.

[33] Chou WH, Huber A, Bentrop J, Schulz S, Schwab K, Chadwell LV, Paulsen R, Britt SG (1999). Patterning of the R7 and R8 photoreceptor cells of drosophila: evidence for induced and default cell-fate specification. Development.

[34] Fortini ME, Rubin GM (1990). Analysis of cis-acting requirements of the Rh3 and Rh4 genes reveals a bipartite organization to rhodopsin promoters in Drosophila Melanogaster. Genes Dev.

[35] Mismer D, Michael WM, Laverty TR, Rubin GM (1988). Analysis of the promoter of the Rh2 opsin gene in Drosophila Melanogaster. Genetics.

[36] Mismer D, Rubin GM (1987). Analysis of the promoter of the ninaE opsin gene in Drosophila Melanogaster. Genetics.

[37] Papatsenko D, Sheng G, Desplan C (1997). A new rhodopsin in R8 photoreceptors of drosophila: evidence for coordinate expression with Rh3 in R7 cells. Development.

[38] Yang Z, Edenberg HJ, Davis RL (2005). Isolation of mRNA from specific tissues of drosophila by mRNA tagging. Nucleic Acids Res.

[39] Ishikawa Y, Kamikouchi A (2016). Auditory system of fruit flies. Hear Res.

[40] Nueda MJ, Tarazona S, Conesa A (2014). Next maSigPro: updating maSigPro bioconductor package for RNA-seq time series. Bioinformatics.

[41] Spies D, Ciaudo C (2015). Dynamics in Transcriptomics: advancements in RNA-seq time course and downstream analysis. Comput Struct Biotechnol J.

[42] Du M, Mangold CA, Bixler GV, Brucklacher RM, Masser DR, Stout MB, Elliott MH, Freeman WM (2017). Retinal gene expression responses to aging are sexually divergent. Mol Vis.

[43] Huang DW, Sherman BT, Lempicki RA (2009). Systematic and integrative analysis of large gene lists using DAVID bioinformatics resources. Nat Protoc.

[44] Chan SH, Yu AM, McVey M (2010). Dual roles for DNA polymerase theta in alternative end-joining repair of double-strand breaks in drosophila. PLoS Genet.

[45] Kane DP, Shusterman M, Rong Y, McVey M (2012). Competition between replicative and translesion polymerases during homologous recombination repair in drosophila. PLoS Genet.

[46] Eeken JC, Romeijn RJ, de Jong AW, Pastink A, Lohman PH (2001). Isolation and genetic characterisation of the drosophila homologue of (SCE)REV3, encoding the catalytic subunit of DNA polymerase zeta. Mutat Res.

[47] Missirlis F, Ulschmid JK, Hirosawa-Takamori M, Gronke S, Schafer U, Becker K, Phillips JP, Jackle H (2002). Mitochondrial and cytoplasmic thioredoxin reductase variants encoded by a single drosophila gene are both essential for viability. J Biol Chem.

[48] Pathak C, Jaiswal YK, Vinayak M (2008). Queuine promotes antioxidant defence system by activating cellular antioxidant enzyme activities in cancer. Biosci Rep.

[49] Hofling C, Indrischek H, Hopcke T, Waniek A, Cynis H, Koch B, Schilling S, Morawski M, Demuth HU, Rossner S (2014). Mouse strain and brain region-specific expression of the glutaminyl cyclases QC and isoQC. Int J Dev Neurosci.

[50] Ognjenovic J, Wu J, Matthies D, Baxa U, Subramaniam S, Ling J, Simonovic M (2016). The crystal structure of human GlnRS provides basis for the development of neurological disorders. Nucleic Acids Res.

[51] Garcia-Huerta P, Bargsted L, Rivas A, Matus S, Vidal RL (2016). ER chaperones in neurodegenerative disease: folding and beyond. Brain Res.

[52] Kubota H, Hynes G, Carne A, Ashworth A, Willison K (1994). Identification of six Tcp-1-related genes encoding divergent subunits of the TCP-1-containing chaperonin. Curr Biol.

[53] Ohtsuka K, Suzuki T (2000). Roles of molecular chaperones in the nervous system. Brain Res Bull.

[54] de Nadal E, Ammerer G, Posas F (2011). Controlling gene expression in response to stress. Nat Rev Genet.

[55] Marash L, Liberman N, Henis-Korenblit S, Sivan G, Reem E, Elroy-Stein O, Kimchi A (2008). DAP5 promotes cap-independent translation of Bcl-2 and CDK1 to facilitate cell survival during mitosis. Mol Cell.

[56] Majzoub K, Hafirassou ML, Meignin C, Goto A, Marzi S, Fedorova A, Verdier Y, Vinh J, Hoffmann JA, Martin F (2014). RACK1 controls IRES-mediated translation of viruses. Cell.

[57] Hall DJ, Grewal SS, de la Cruz AF, Edgar BA (2007). Rheb-TOR signaling promotes protein synthesis, but not glucose or amino acid import, in drosophila. BMC Biol.

[58] Johnson SC, Rabinovitch PS, Kaeberlein M (2013). mTOR is a key modulator of ageing and age-related disease. Nature.

[59] Juusola M, Niven JE, French AS (2003). Shaker K+ channels contribute early nonlinear amplification to the light response in drosophila photoreceptors. J Neurophysiol.

[60] Vahasoyrinki M, Niven JE, Hardie RC, Weckstrom M, Juusola M (2006). Robustness of neural coding in drosophila photoreceptors in the absence of slow delayed rectifier K+ channels. J Neurosci.

[61] Palladino MJ, Bower JE, Kreber R, Ganetzky B (2003). Neural dysfunction and neurodegeneration in drosophila Na+/K+ ATPase alpha subunit mutants. J Neurosci.

[62] Hardie RC (1991). Voltage-sensitive potassium channels in drosophila photoreceptors. J Neurosci.

[63] Bronk P, Nie Z, Klose MK, Dawson-Scully K, Zhang J, Robertson RM, Atwood HL, Zinsmaier KE (2005). The multiple functions of cysteine-string protein analyzed at drosophila nerve terminals. J Neurosci.

[64] Frolov RV, Bagati A, Casino B, Singh S (2012). Potassium channels in drosophila: historical breakthroughs, significance, and perspectives. J Neurogenet.

[65] Saumweber T, Weyhersmuller A, Hallermann S, Diegelmann S, Michels B, Bucher D, Funk N, Reisch D, Krohne G, Wegener S (2011). Behavioral and synaptic plasticity are impaired upon lack of the synaptic protein SAP47. J Neurosci.

[66] Burg MG, Sarthy PV, Koliantz G, Pak WL (1993). Genetic and molecular identification of a drosophila histidine decarboxylase gene required in photoreceptor transmitter synthesis. EMBO J.

[67] Weiss S, Kohn E, Dadon D, Katz B, Peters M, Lebendiker M, Kosloff M, Colley NJ, Minke B (2012). Compartmentalization and Ca2+ buffering are essential for prevention of light-induced retinal degeneration. J Neurosci.

[68] Ballinger DG, Xue N, Harshman KD (1993). A drosophila photoreceptor cell-specific protein, calphotin, binds calcium and contains a leucine zipper. Proc Natl Acad Sci U S A.

[69] Martin JH, Benzer S, Rudnicka M, Miller CA (1993). Calphotin: a drosophila photoreceptor cell calcium-binding protein. Proc Natl Acad Sci U S A.

[70] Jeong K, Lee S, Seo H, Oh Y, Jang D, Choe J, Kim D, Lee JH, Jones WD (2015). Ca-alpha1T, a fly T-type Ca2+ channel, negatively modulates sleep. Sci Rep.

[71] Rosenbaum EE, Vasiljevic E, Brehm KS, Colley NJ (2014). Mutations in four glycosyl hydrolases reveal a highly coordinated pathway for rhodopsin biosynthesis and N-glycan trimming in Drosophila Melanogaster. PLoS Genet.

[72] Han J, Reddig K, Li HS (2007). Prolonged G(q) activity triggers fly rhodopsin endocytosis and degradation, and reduces photoreceptor sensitivity. EMBO J.

[73] O'Tousa JE (1992). Requirement of N-linked glycosylation site in drosophila rhodopsin. Vis Neurosci.

[74] Brown G, Chen DM, Christianson JS, Lee R, Stark WS (1994). Receptor demise from alteration of glycosylation site in drosophila opsin: electrophysiology, microspectrophotometry, and electron microscopy. Vis Neurosci.

[75] Katanosaka K, Tokunaga F, Kawamura S, Ozaki K (1998). N-linked glycosylation of drosophila rhodopsin occurs exclusively in the amino-terminal domain and functions in rhodopsin maturation. FEBS Lett.

[76] Hall JC, Alahiotis SN, Strumpf DA, White K (1980). Behavioral and biochemical defects in temperature-sensitive acetylcholinesterase mutants of Drosophila Melanogaster. Genetics.

[77] Perrimon N, Smouse D, Miklos GLG (1989). Developmental genetics of loci at the base of the X-chromosome of drosophila-Melanogaster. Genetics.

[78] Dimitroff B, Howe K, Watson A, Campion B, Lee HG, Zhao N, O'Connor MB, Neufeld TP, Selleck SB (2012). Diet and energy-sensing inputs affect TorC1-mediated axon misrouting but not TorC2-directed synapse growth in a drosophila model of tuberous sclerosis. PLoS One.

[79] Mount SM, Salz HK (2000). Pre-messenger RNA processing factors in the drosophila genome. J Cell Biol.

[80] Park JW, Parisky K, Celotto AM, Reenan RA, Graveley BR (2004). Identification of alternative splicing regulators by RNA interference in drosophila. Proc Natl Acad Sci U S A.

[81] Juge F, Zaessinger S, Temme C, Wahle E, Simonelig M (2002). Control of poly(a) polymerase level is essential to cytoplasmic polyadenylation and early development in drosophila. EMBO J.

[82] Okamura K, Ishizuka A, Siomi H, Siomi MC (2004). Distinct roles for Argonaute proteins in small RNA-directed RNA cleavage pathways. Genes Dev.

[83] Wei W, Ba Z, Gao M, Wu Y, Ma Y, Amiard S, White CI, Rendtlew Danielsen JM, Yang YG, Qi Y (2012). A role for small RNAs in DNA double-strand break repair. Cell.

[84] Freeman A, Franciscovich A, Bowers M, Sandstrom DJ, Sanyal S (2011). NFAT regulates pre-synaptic development and activity-dependent plasticity in drosophila. Mol Cell Neurosci.

[85] Nguyen DNT, Rohrbaugh M, Lai ZC (2000). The drosophila homolog of Onecut homeodomain proteins is a neural-specific transcriptional activator with a potential role in regulating neural differentiation. Mech Develop.

[86] Ryoo HD, Domingos PM, Kang MJ, Steller H (2007). Unfolded protein response in a drosophila model for retinal degeneration. EMBO J.

[87] Mohan M, Herz HM, Smith ER, Zhang Y, Jackson J, Washburn MP, Florens L, Eissenberg JC, Shilatifard A (2011). The COMPASS family of H3K4 methylases in drosophila. Mol Cell Biol.

[88] Aoyagi N, Wassarman DA (2000). Genes encoding Drosophila Melanogaster RNA polymerase II general transcription factors: diversity in TFIIA and TFIID components contributes to gene-specific transcriptional regulation. J Cell Biol.

[89] Heinz S, Benner C, Spann N, Bertolino E, Lin YC, Laslo P, Cheng JX, Murre C, Singh H, Glass CK (2010). Simple combinations of lineage-determining transcription factors prime cis-regulatory elements required for macrophage and B cell identities. Mol Cell.

[90] Nitta KR, Jolma A, Yin Y, Morgunova E, Kivioja T, Akhtar J, Hens K, Toivonen J, Deplancke B, Furlong EE, et al. Conservation of transcription factor binding specificities across 600 million years of bilateria evolution. eLife 2015;4:e04837. doi:10.7554/eLife.04837. 10.7554/eLife.04837 PMC4362205 25779349

[91] Vandoren M, Bailey AM, Esnayra J, Ede K, Posakony JW (1994). Negative regulation of proneural gene activity - hairy is a direct transcriptional repressor of Achaete. Genes Dev.

[92] Perkins KK, Admon A, Patel N, Tjian R (1990). The drosophila Fos-related Ap-1 protein is a developmentally regulated transcription factor. Genes Dev.

[93] Luo X, Puig O, Hyun J, Bohmann D, Jasper H (2007). Foxo and Fos regulate the decision between cell death and survival in response to UV irradiation. EMBO J.

[94] Gabel HW, Kinde B, Stroud H, Gilbert CS, Harmin DA, Kastan NR, Hemberg M, Ebert DH, Greenberg ME (2015). Disruption of DNA-methylation-dependent long gene repression in Rett syndrome. Nature.

[95] Mohr C, Hartmann B (2014). Alternative splicing in Drosophila neuronal development. J Neurogenet.

[96] King IF, Yandava CN, Mabb AM, Hsiao JS, Huang HS, Pearson BL, Calabrese JM, Starmer J, Parker JS, Magnuson T (2013). Topoisomerases facilitate transcription of long genes linked to autism. Nature.

[97] Vermeij WP, Dollé MET, Reiling E, Jaarsma D, Payan-Gomez C, Bombardieri CR, Wu H, Roks AJM, Botter SM, van der Eerden BC, Youssef SA, Kuiper RV, Nagarajah B, van Oostrom CT, Brandt RMC, Barnhoorn S, Imholz S, Pennings JLA, de Bruin A, Gyenis Á, Pothof J, Vijg J, van Steeg H, Hoeijmakers JHJ. Restricted diet delays accelerated ageing and genomic stress in DNA-repair-deficient mice. Nature. 2016;537:427–31. doi:10.1038/nature19329. 10.1038/nature19329 PMC5161687 27556946

[98] Stegeman R, Weake VM. Transcriptional signatures of aging. J Mol Biol. 2017; 10.1016/j.jmb.2017.06.019 PMC5662117 28684248

[99] Conesa A, Madrigal P, Tarazona S, Gomez-Cabrero D, Cervera A, McPherson A, Szczesniak MW, Gaffney DJ, Elo LL, Zhang X (2016). A survey of best practices for RNA-seq data analysis. Genome Biol.

[100] Westholm Jakub O, Miura P, Olson S, Shenker S, Joseph B, Sanfilippo P, Celniker Susan E, Graveley Brenton R, Lai Eric C (2014). Genome-wide analysis of drosophila circular RNAs reveals their structural and sequence properties and age-dependent neural accumulation. Cell Rep.

[101] Ashwal-Fluss R, Meyer M, Pamudurti NR, Ivanov A, Bartok O, Hanan M, Evantal N, Memczak S, Rajewsky N (2014). Kadener S: **circRNA biogenesis competes with pre-mRNA splicing**. Mol Cell.

[102] Gao Y, Wang J, Zhao F (2015). CIRI: an efficient and unbiased algorithm for de novo circular RNA identification. Genome Biol.

[103] Gao Y, Zhang J, Zhao F. Circular RNA identification based on multiple seed matching. Brief Bioinform. 2017;bbx014. https://doi.org/10.1093/bib/bbx014. 10.1093/bib/bbx014 28334140

[104] Gruner H, Cortes-Lopez M, Cooper DA, Bauer M, Miura P (2016). CircRNA accumulation in the aging mouse brain. Sci Rep.

[105] Bishop NA, Lu T, Yankner BA (2010). Neural mechanisms of ageing and cognitive decline. Nature.

[106] Chowers I, Liu D, Farkas RH, Gunatilaka TL, Hackam AS, Bernstein SL, Campochiaro PA, Parmigiani G, Zack DJ (2003). Gene expression variation in the adult human retina. Hum Mol Genet.

[107] Sharon D, Blackshaw S, Cepko CL, Dryja TP (2002). Profile of the genes expressed in the human peripheral retina, macula, and retinal pigment epithelium determined through serial analysis of gene expression (SAGE). Proc Natl Acad Sci U S A.

[108] Lu T, Pan Y, Kao SY, Li C, Kohane I, Chan J, Yankner BA (2004). Gene regulation and DNA damage in the ageing human brain. Nature.

[109] Loerch PM, Lu T, Dakin KA, Vann JM, Isaacs A, Geula C, Wang J, Pan Y, Gabuzda DH, Li C (2008). Evolution of the aging brain transcriptome and synaptic regulation. PLoS One.

[110] Berchtold NC, Cribbs DH, Coleman PD, Rogers J, Head E, Kim R, Beach T, Miller C, Troncoso J, Trojanowski JQ (2008). Gene expression changes in the course of normal brain aging are sexually dimorphic. Proc Natl Acad Sci U S A.

[111] Girardot F, Lasbleiz C, Monnier V, Tricoire H (2006). Specific age-related signatures in drosophila body parts transcriptome. BMC Genomics.

[112] Kuintzle RC, Chow ES, Westby TN, Gvakharia BO, Giebultowicz JM, Hendrix DA (2017). Circadian deep sequencing reveals stress-response genes that adopt robust rhythmic expression during aging. Nat Commun.

[113] Lee SJ, Feldman R, O'Farrell PH (2008). An RNA interference screen identifies a novel regulator of target of Rapamycin that mediates hypoxia suppression of translation in drosophila S2 cells. Mol Biol Cell.

[114] Zhou B, Williams DW, Altman J, Riddiford LM, Truman JW (2009). Temporal patterns of broad isoform expression during the development of neuronal lineages in drosophila. Neural Dev.

[115] Armstrong JD, Texada MJ, Munjaal R, Baker DA, Beckingham KM (2006). Gravitaxis in Drosophila Melanogaster: a forward genetic screen. Genes Brain Behav.

[116] Kim SN, Rhee JH, Song YH, Park DY, Hwang M, Lee SL, Kim JE, Gim BS, Yoon JH, Kim YJ (2005). Age-dependent changes of gene expression in the drosophila head. Neurobiol Aging.

[117] Neretti N, Wang P-Y, Brodsky AS, Nyguyen HH, White KP, Rogina B, Helfand SL (2009). Long-lived Indy induces reduced mitochondrial reactive oxygen species production and oxidative damage. Proc Natl Acad Sci.

[118] Wood JG, Jones BC, Jiang N, Chang C, Hosier S, Wickremesinghe P, Garcia M, Hartnett DA, Burhenn L, Neretti N (2016). Chromatin-modifying genetic interventions suppress age-associated transposable element activation and extend life span in drosophila. Proc Natl Acad Sci U S A.

[119] Doyle JP, Dougherty JD, Heiman M, Schmidt EF, Stevens TR, Ma G, Bupp S, Shrestha P, Shah RD, Doughty ML (2008). Application of a translational profiling approach for the comparative analysis of CNS cell types. Cell.

[120] Heiman M, Kulicke R, Fenster RJ, Greengard P, Heintz N (2014). Cell type-specific mRNA purification by translating ribosome affinity purification (TRAP). Nat Protoc.

[121] Lewis EB (1960). A new standard food medium. Drosophila Information Service.

[122] Tully T, Quinn WG (1985). Classical conditioning and retention in normal and mutant Drosophila Melanogaster. J Comp Physiol A.

[123] Bustin SA, Benes V, Garson JA, Hellemans J, Huggett J, Kubista M, Mueller R, Nolan T, Pfaffl MW, Shipley GL (2009). The MIQE guidelines: minimum information for publication of quantitative real-time PCR experiments. Clin Chem.

[124] Robinson MD, McCarthy DJ, Smyth GK (2010). edgeR: a Bioconductor package for differential expression analysis of digital gene expression data. Bioinformatics.

[125] Alexa A, Rahnenfuhrer J. topGO: enrichment analysis for gene ontology. R Package Version. 2016;2260.https://bioconductor.org/packages/release/bioc/html/topGO.html.

[126] Robin X, Turck N, Hainard A, Tiberti N, Lisacek F, Sanchez JC, Muller M (2011). pROC: an open-source package for R and S+ to analyze and compare ROC curves. BMC Bioinformatics.

[127] Montojo J, Zuberi K, Rodriguez H, Bader GD, Morris Q (2014). GeneMANIA: fast gene network construction and function prediction for Cytoscape. F1000Res.

[128] Quinlan AR, Hall IM (2010). BEDTools: a flexible suite of utilities for comparing genomic features. Bioinformatics.

[129] Liao Y, Smyth GK, Shi W (2014). featureCounts: an efficient general purpose program for assigning sequence reads to genomic features. Bioinformatics.

