# Supplementary material for: Transcriptome profiling of aging Drosophila photoreceptors reveals gene expression trends that correlate with visual senescence
Source: BMC Genomics. 2017 Nov 21;18:894. doi: 10.1186/s12864-017-4304-3 (PMC5698953; doi:10.1186/s12864-017-4304-3)
Supplement: Supplementary file 1 — Complete Supplemental Figures. Figure S1. Affinity-purified nuclear RNA is enriched for photoreceptor-expressed genes. Figure S2. Rh1-Gal4 drives GFP expression in antennal sensory neurons. Figure S3. Relative sensory neuron proportions and yields of affinity-purified nuclear RNA do not change with age. Figure S4. qPCR of selected age-regulated genes. Figure S5. K-means clustering of age-regulated genes based on temporal expression pattern. Figure S6. Top promoter motifs that predict age-related expression changes. Figure S7. Promoter motifs with the best predictive power co-occur frequently with a variety of other motifs. Figure S8. Distribution of the top motifs in age upregulated genes. Figure S9. Distribution of the top motifs in age upregulated genes. Figure S10. Distribution of the top motifs between expression clusters. Figure S11. Downregulated gene clusters are enriched for longer, more highly expressed and more heavily spliced genes. Figure S12. qPCR of selected age-regulated circRNAs. Figure S13. circRNA-containing host genes are enriched for longer and more heavily spliced genes. (PDF 4807 kb) [file 12864_2017_4304_MOESM1_ESM.pdf]

SUPPLEMENTAL FIGURE LEGENDS:

Figure S1: Affinity-purified nuclear RNA is enriched for photoreceptor-expressed genes. (A) Head homogenates were prepared from equal numbers of flies expressing KASH-GFP or KASH-mCherry under Rh1-Gal4 control, generating a mixture with equal numbers of GFP- and mCherry-labeled nuclei. KASH-GFP nuclei were affinity-enriched, and GFP and mCherry mRNA levels were measured in the pre- and post-isolation samples by qPCR. The expression of each gene was normalized to the geometric mean of two reference genes (*eIF1A* and *RpL32*) and is shown relative to the pre-isolation sample, which is set to one. Data represent mean ± standard deviation (s.d.; n = 3). (B) Principal component analysis for pre- and post-isolation RNA-seq samples from day 10 male flies based on counts per million (CPM). (C) Volcano plot showing the fold change post-enrichment plotted as $\log_2$(fold change) for each gene relative to its false discovery rate ($-\log_2$[FDR]). Genes with significantly differential expression (FDR < 0.05 and FC > 2, dotted lines) are highlighted in red or blue. (D) Significantly-enriched GO terms ($p < 0.0001$, Fisher's test) were identified for 447 post-enriched genes or 444 post-reduced genes relative to all 5261 expressed genes using TopGO. Similar GO terms were grouped based on intersecting gene members, and the percentage of expressed genes with each functional annotation that were post-enriched or reduced was displayed on the bar plot. The number of genes in each GO term group is shown to the right of each bar in italics. Genes with the indicated GO terms identified in the post-enriched or reduced gene sets are shown in the inset boxes for selected GO terms.

Figure S2: Rh1-Gal4 drives GFP expression in antennal sensory neurons. Bar plot showing the relative expression of each indicated gene in different tissues normalized to *RpL32* levels, with head expression set to one. Cq values for each gene and tissue are shown above each bar. *nompA* gene is an auditory gene expressed in the Johnston's organ in antennae and *Cpn* is an eye-specific gene expressed in photoreceptors.

Figure S3: Relative sensory neuron proportions and yields of affinity-purified nuclear RNA do not change with age. (A) Bar plot showing the relative RNA yield from KASH-GFP affinity-purifications at each age as mean ± s.d. (n = 3). *p* values, Student's *t*-test between each age and day 10. (B) Bar plot showing the relative expression of each indicated gene in dissected eyes from *Rh1-Gal4>KASH-GFP* flies at day 10 and day 40. The expression of each gene was normalized to the geometric mean of two reference genes (*eIF1A* and *ninaA*) and is shown relative to day 10, which is set to one. Data represent mean ± s.d. (n = 3). *p* values, Student's *t*-test between ages. ns, not significant. (C) Western blot showing GFP levels in dissected eyes from day 10 and day 40 flies. (D) Heatmap showing relative mean expression across the indicated ages of genes with the GO term GO:0007602 (phototransduction) and/or GO:0007605 (sensory perception of sound). Genes that are expressed only in antennae based on published reports are shown in the upper panel (antennae-specific).

Figure S4: qPCR of selected age-regulated genes. Bar plots showing qPCR analysis of selected age-regulated genes in day 10 and day 40 flies. Transcript levels were measured by qPCR in dissected eyes from male flies (panel A), independent affinity-enriched photoreceptor nuclear RNA samples from male flies (panel B), or dissected eyes from female flies (panel C). The expression of each gene was normalized to the geometric mean of two reference genes (*eIF1A* and *ninaA*) and is shown relative to day 10, which is set to one. Data represent mean ± s.d. (n = 3). *p* values are shown for significant comparisons ($p < 0.05$) based on Student's *t*-test between ages. Three of the tested genes (*Hsc70-4*, *Ku80*, and *rad50*) were not present at high enough levels in both time points in either male eyes or affinity-enriched photoreceptor nuclear RNA to ensure reproducible qPCR data, and were excluded from the respective bar plot.

Figure S5: K-means clustering of age-regulated genes based on temporal expression pattern. (A) Age-regulated genes (555 genes) were separated into 1 – 20 clusters using k-

means clustering based on their mean relative expression at each time point. Linear or polynomial models were fitted to the mean relative expression of all genes in each cluster versus age, and the residual standard errors and adjacent r^2 values were determined for each cluster. Scatter plots show the residual standard errors (left panel) and adjacent r^2 values (right panel) summed across all clusters for k-means clustering using 1 to 20 clusters for linear, second degree and third degree polynomial models. Arrows indicate the number of clusters (11) selected for k-means clustering shown in Figure 2. (B) Line graphs showing the derivatives of the fitted curves (second degree polynomials) for each cluster shown in Figure 2. Age-regulated genes in each cluster were designated as up or downregulated, and early, middle or late as shown.

Figure S6: Top promoter motifs that predict age-related expression changes. The top 14 sequence motifs identified for down (left panels) or up-regulated (right panels) genes. The top motif for each group is shown in the left column, top row (i.e. motif 2 downregulated genes, motif 1 upregulated genes). Motif numbers are based on the initial HOMER *p* values for enrichment in target versus background gene sets (Table S5). Putative transcription factors that bind each motif and respective scores are shown for the highest scoring expressed candidate transcription factor. Table S6 shows a full list of expressed candidate transcription factors that could bind each motif. The percentage of genes in the target gene set (T:%) and *p*-value for each motif are shown (enrichment in target versus background). If the highest scoring expressed candidate transcription factor shows differential expression with age, its respective expression cluster is shown (*eg* C1) corresponding to the expression clusters described in Figure 2.

Figure S7: Promoter motifs with the best predictive power co-occur frequently with a variety of other motifs. (A) Network analysis of co-occuring down- or upregulated promoter motifs presented using Fruchterman-Reingold layout algorithm. Nodes represent motifs and

connecting lines (edges) represent co-occurrence frequency at gene promoters. The top 14 motifs with predictive power are indicated as filled blue or red circles respectively. (B) The degree (number of edges per motif) and clustering coefficient (probability that adjacent nodes are connected) are shown for the motifs identified for down- or upregulated genes. Motifs are ordered based on predictive power as described in Figure 3 with the top 14 motifs shown on the left of the panel.

Figure S8: Distribution of the top motifs in age upregulated genes. Heatmap showing the presence of the indicated top sequence motif (columns) in the promoter of age upregulated genes (rows). 114 of the 288 upregulated genes have at least one of the top 14 motifs in their promoters. The expression cluster of each gene based on Figure 2 is shown by the colored panel on the left. Motifs and genes were grouped using hierarchical clustering to show groups of genes with common combinations of the top motifs.

Figure S9: Distribution of the top motifs in age upregulated genes. Heatmap showing the presence of the indicated top sequence motif (columns) in the promoter of age downregulated genes (rows). 124 of the 267 downregulated genes have at least one of the top 14 motifs in their promoters. The expression cluster of each gene based on Figure 2 is shown by the colored panel on the left. Motifs and genes were grouped using hierarchical clustering to show groups of genes with common combinations of the top motifs.

Figure S10: Distribution of the top motifs between expression clusters. Bar plots showing the fraction of genes in each expression cluster (see Figure 2) with the indicated motif for the upregulated and downregulated genes. Motifs are shown in order based on increasing indicative power as shown in Figure 3B.

Figure S11: Downregulated gene clusters are enriched for longer, more highly expressed and more heavily spliced genes. Box plots showing gene length including introns, expression (RPKM), number of expressed exons and transcripts isoforms for genes in the 11 expression clusters relative to genes that are not age regulated (nonsig). Cluster number corresponds to Figure 2. Lower and upper hinges correspond to the first and third quartiles, and the whiskers extend to the smallest or largest values no more than 1.5 x inter-quartile range from each hinge. Some upper outliers on the exon and transcript boxplots fall outside of the range used to display the data. The distribution of genes in each cluster was compared with nonsignificant genes using pairwise Wilcoxon Rank Sum Test with Benjamini and Hochberg correction, and the FDR for significant comparisons is shown above the respective boxplots.

Figure S12: qPCR of selected age-regulated circRNAs. Bar plots showing qPCR analysis of selected circRNAs in independent affinity-enriched photoreceptor nuclear RNA from male day 10 and day 40 flies. The expression of each circRNA was normalized to the geometric mean of two reference genes (*eIF1A* and *ninaA*) and is shown relative to day 10, which is set to one. Data represents mean ± s.d. (n = 3). *p* values, Student's *t*-test between ages. ns, not significant.

Figure S13: circRNA-containing host genes are enriched for longer and more heavily spliced genes. Box plots showing gene length including introns, number of expressed exons and transcripts isoforms for 218 unique genes corresponding to 315 abundant circRNAs (>6 counts total) versus all other expressed genes. Lower and upper hinges correspond to the first and third quartiles, and the whiskers extend to the smallest or largest values no more than 1.5 x inter-quartile range from each hinge. Some upper outliers on the exon and transcript boxplots fall outside of the range used to display the data. The distribution of circRNA genes was compared with all other genes using pairwise Wilcoxon Rank Sum Test with Benjamini and Hochberg correction, and the FDR for significant comparisons is shown.

Hall_FigS1

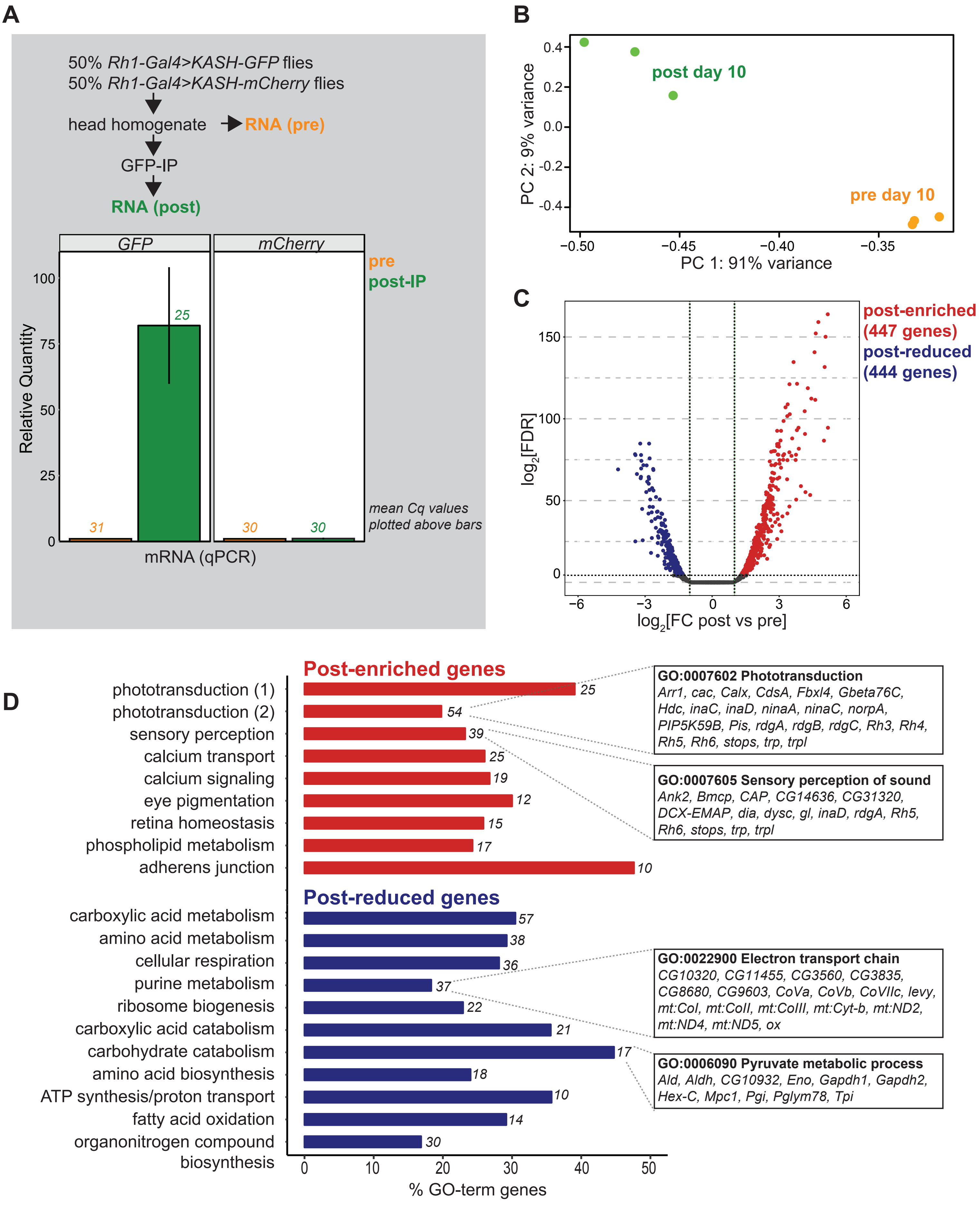
A
50% Rh1-Gal4>KASH-GFP flies
50% Rh1-Gal4>KASH-mCherry flies
head homogenate
RNA (pre)
GFP-IP
RNA (post)
GFP
mCherry
pre
post-IP
Relative Quantity
25
31
30
30
mean Cq values plotted above bars
mRNA (qPCR)
B
post day 10
pre day 10
PC 2: 9% variance
PC 1: 91% variance
C
post-enriched (447 genes)
post-reduced (444 genes)
log2[FDR]
log2[FC post vs pre]
D
Post-enriched genes
phototransduction (1)
phototransduction (2)
sensory perception
calcium transport
calcium signaling
eye pigmentation
retina homeostasis
phospholipid metabolism
adherens junction
25
54
39
25
19
12
15
17
10
GO:0007602 Phototransduction
Arr1, cac, Calx, CdsA, Fbxl4, Gbeta76C, Hdc, inaC, inaD, ninaA, ninaC, norpA, PIP5K59B, Pis, rdgA, rdgB, rdgC, Rh3, Rh4, Rh5, Rh6, stops, trp, trpl
GO:0007605 Sensory perception of sound
Ank2, Bmcp, CAP, CG14636, CG31320, DCX-EMAP, dia, dysc, gl, inaD, rdgA, Rh5, Rh6, stops, trp, trpl
Post-reduced genes
carboxylic acid metabolism
amino acid metabolism
cellular respiration
purine metabolism
ribosome biogenesis
carboxylic acid catabolism
carbohydrate catabolism
amino acid biosynthesis
ATP synthesis/proton transport
fatty acid oxidation
organonitrogen compound biosynthesis
57
38
36
37
22
21
17
18
10
14
30
GO:0022900 Electron transport chain
CG10320, CG11455, CG3560, CG3835, CG8680, CG9603, CoVa, CoVb, CoVIIc, levy, mt:CoI, mt:CoII, mt:CoIII, mt:Cyt-b, mt:ND2, mt:ND4, mt:ND5, ox
GO:0006090 Pyruvate metabolic process
Ald, Aldh, CG10932, Eno, Gapdh1, Gapdh2, Hex-C, Mpc1, Pgi, Pglym78, Tpi
% GO-term genes

Hall_FigS2

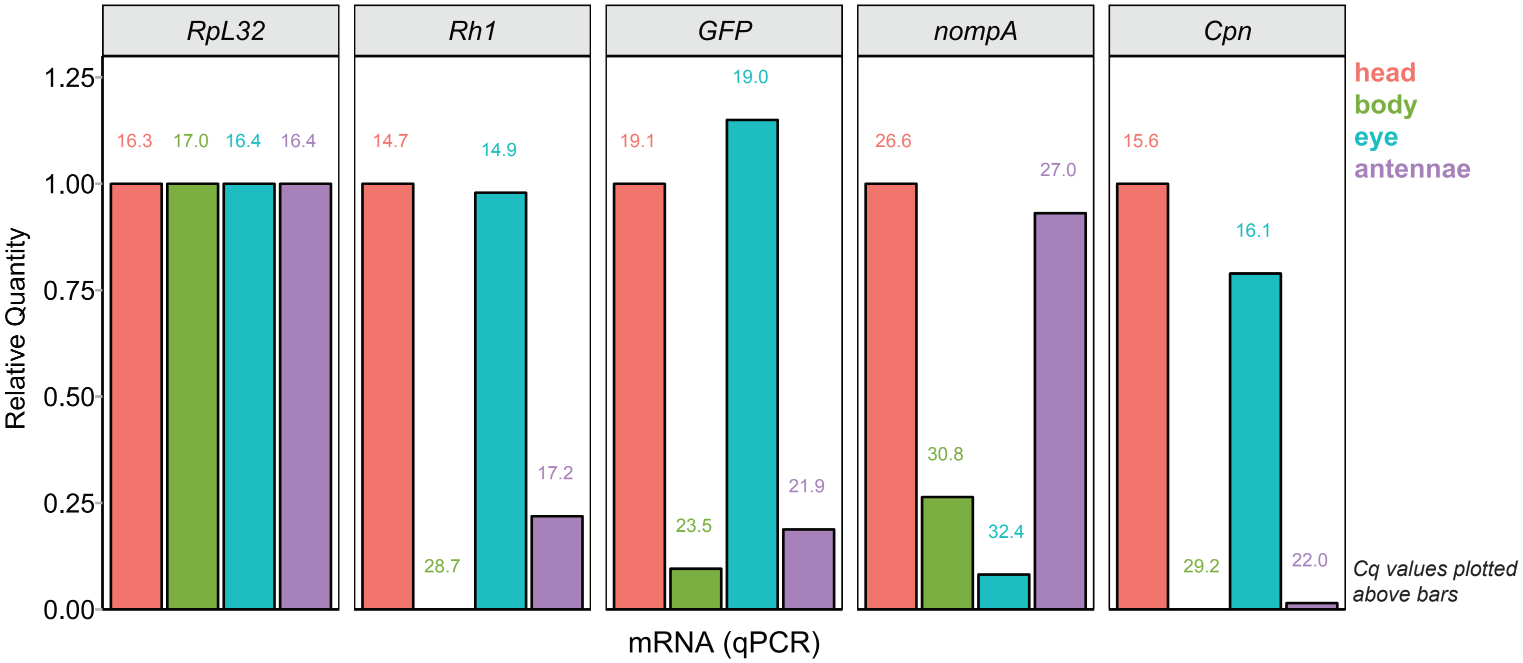
RpL32
Rh1
GFP
nompA
Cpn
Relative Quantity
1.25
1.00
0.75
0.50
0.25
0.00
16.3
17.0
16.4
16.4
14.7
28.7
14.9
17.2
19.1
23.5
19.0
21.9
26.6
30.8
32.4
27.0
15.6
29.2
16.1
22.0
head
body
eye
antennae
Cq values plotted
above bars
mRNA (qPCR)

Hall_FigS3

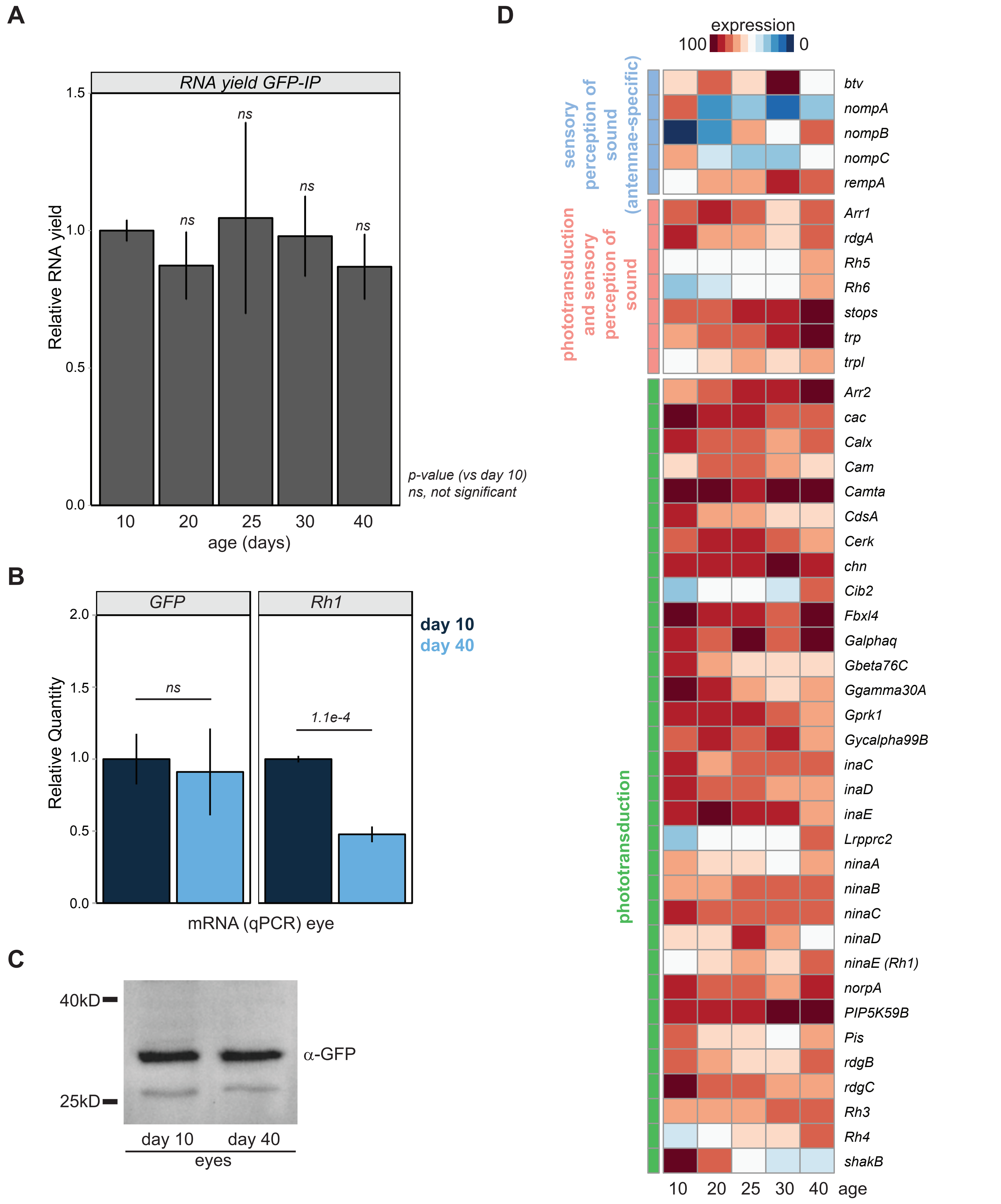

A
RNA yield GFP-IP
Relative RNA yield
ns
p-value (vs day 10)
ns, not significant
10 20 25 30 40
age (days)
B
GFP
Rh1
Relative Quantity
ns
1.1e-4
day 10
day 40
mRNA (qPCR) eye
C
40kD
25kD
α-GFP
day 10 day 40
eyes
D
expression
100 0
sensory perception of sound (antennae-specific)
phototransduction and sensory perception of sound
phototransduction
btv
nompA
nompB
nompC
rempA
Arr1
rdgA
Rh5
Rh6
stops
trp
trpl
Arr2
cac
Calx
Cam
Camta
CdsA
Cerk
chn
Cib2
Fbxl4
Galphaq
Gbeta76C
Ggamma30A
Gprk1
Gycalpha99B
inaC
inaD
inaE
Lrpprc2
ninaA
ninaB
ninaC
ninaD
ninaE (Rh1)
norpA
PIP5K59B
Pis
rdgB
rdgC
Rh3
Rh4
shakB
10 20 25 30 40 age

Hall_FigS4

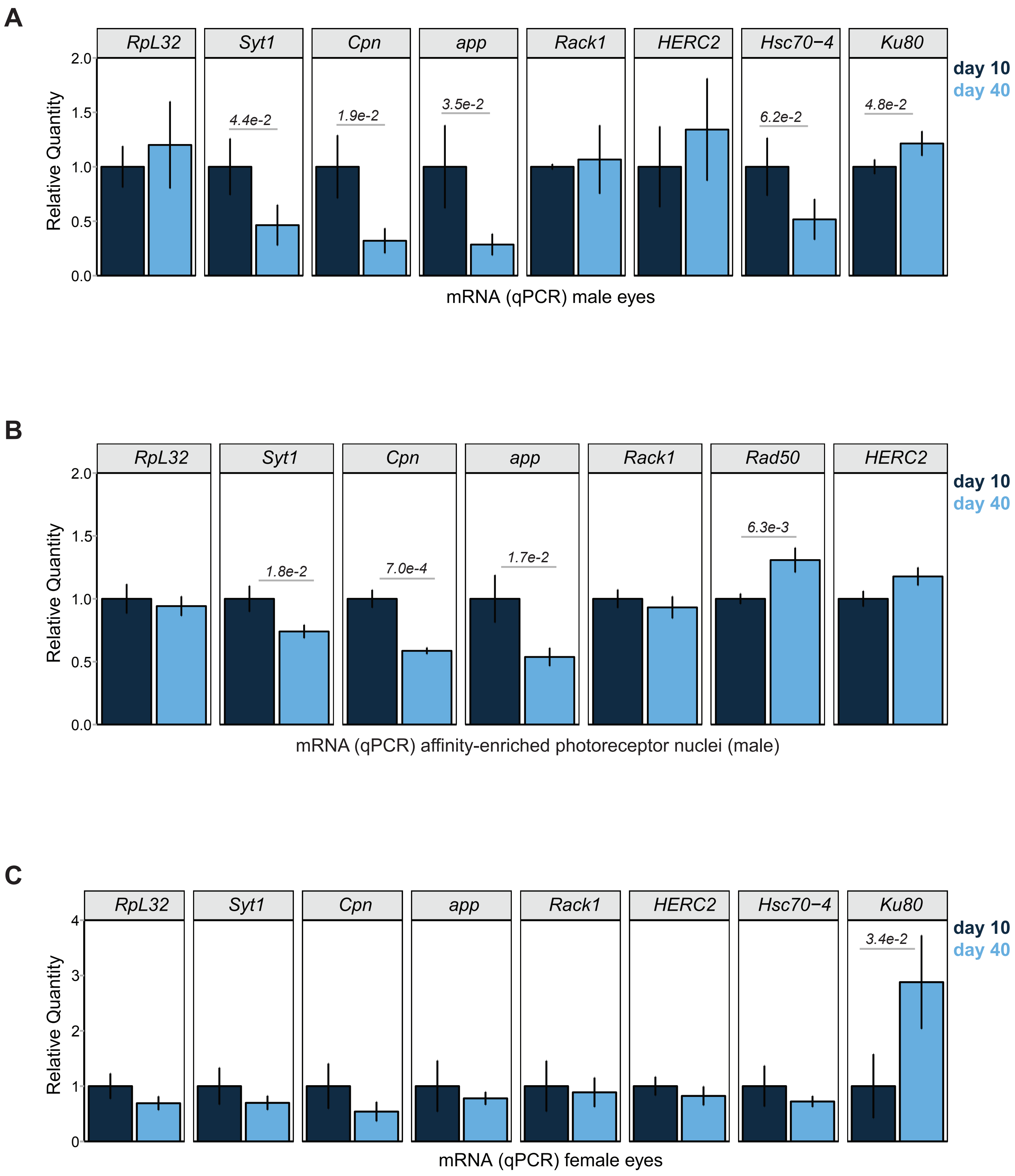
A
RpL32
Syt1
Cpn
app
Rack1
HERC2
Hsc70−4
Ku80
Relative Quantity
0.0
0.5
1.0
1.5
2.0
4.4e-2
1.9e-2
3.5e-2
6.2e-2
4.8e-2
day 10
day 40
mRNA (qPCR) male eyes
B
RpL32
Syt1
Cpn
app
Rack1
Rad50
HERC2
Relative Quantity
1.8e-2
7.0e-4
1.7e-2
6.3e-3
day 10
day 40
mRNA (qPCR) affinity-enriched photoreceptor nuclei (male)
C
RpL32
Syt1
Cpn
app
Rack1
HERC2
Hsc70−4
Ku80
Relative Quantity
0
1
2
3
4
3.4e-2
day 10
day 40
mRNA (qPCR) female eyes

Hall_FigS5

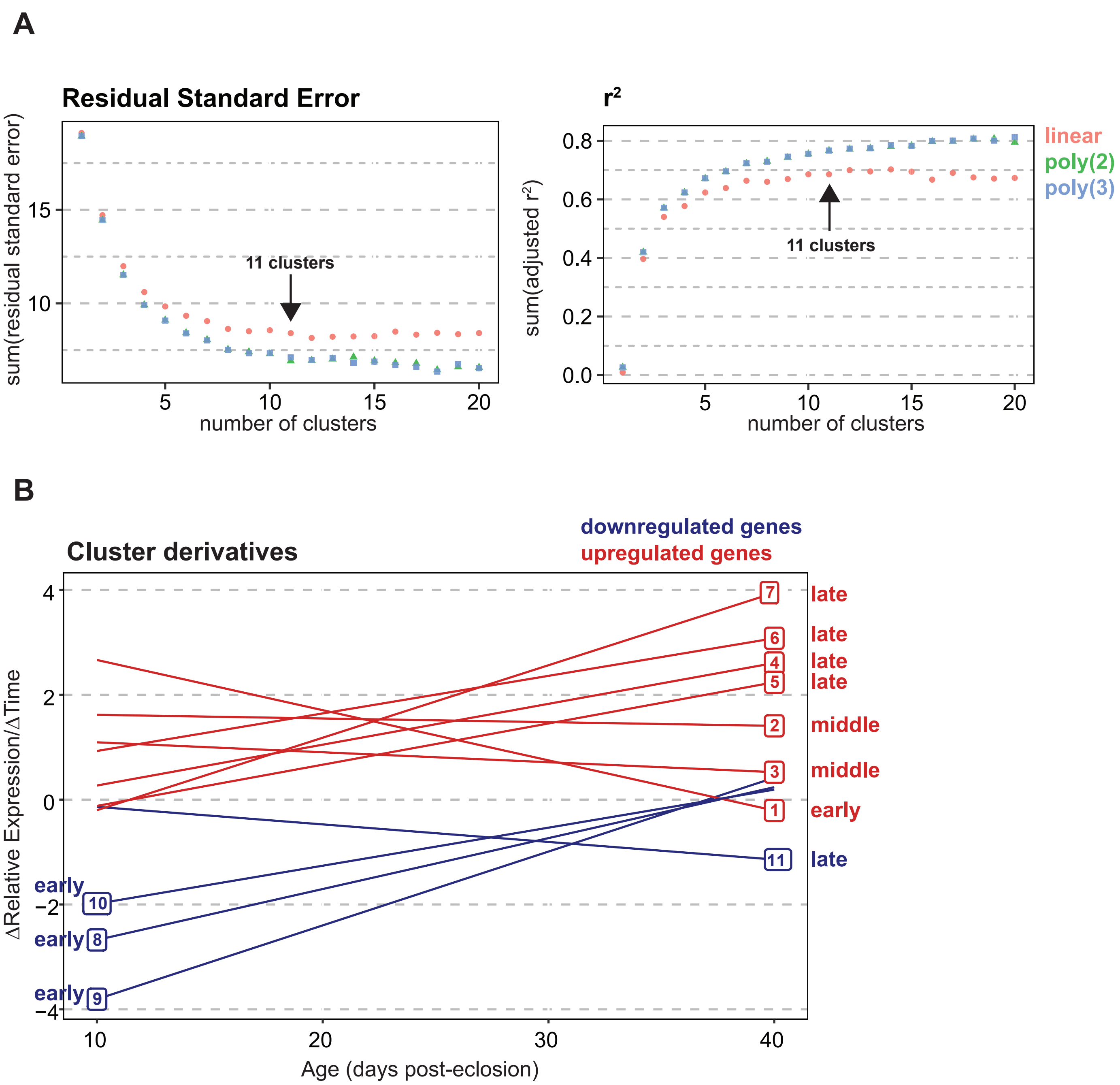
A
Residual Standard Error
sum(residual standard error)
11 clusters
number of clusters
r²
sum(adjusted r²)
11 clusters
number of clusters
linear
poly(2)
poly(3)
B
Cluster derivatives
downregulated genes
upregulated genes
ΔRelative Expression/ΔTime
Age (days post-eclosion)
late
late
late
late
middle
middle
early
late
early
early
early

Hall_FigS6

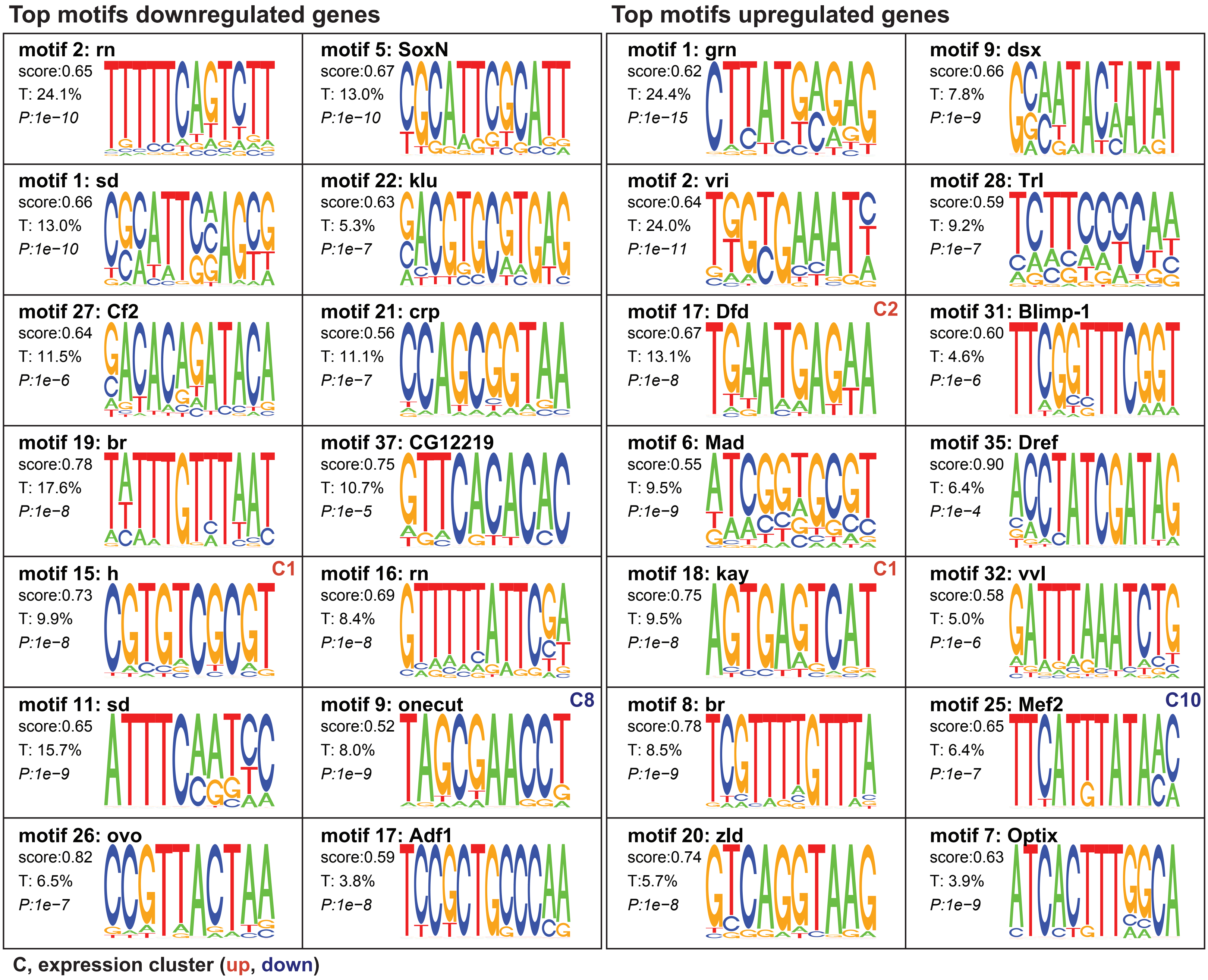

Top motifs downregulated genes
motif 2: rn
score:0.65
T: 24.1%
P:1e−10
motif 5: SoxN
score:0.67
T: 13.0%
P:1e−10
motif 1: sd
score:0.66
T: 13.0%
P:1e−10
motif 22: klu
score:0.63
T: 5.3%
P:1e−7
motif 27: Cf2
score:0.64
T: 11.5%
P:1e−6
motif 21: crp
score:0.56
T: 11.1%
P:1e−7
motif 19: br
score:0.78
T: 17.6%
P:1e−8
motif 37: CG12219
score:0.75
T: 10.7%
P:1e−5
motif 15: h
C1
score:0.73
T: 9.9%
P:1e−8
motif 16: rn
score:0.69
T: 8.4%
P:1e−8
motif 11: sd
score:0.65
T: 15.7%
P:1e−9
motif 9: onecut
C8
score:0.52
T: 8.0%
P:1e−9
motif 26: ovo
score:0.82
T: 6.5%
P:1e−7
motif 17: Adf1
score:0.59
T: 3.8%
P:1e−8
Top motifs upregulated genes
motif 1: grn
score:0.62
T: 24.4%
P:1e−15
motif 9: dsx
score:0.66
T: 7.8%
P:1e−9
motif 2: vri
score:0.64
T: 24.0%
P:1e−11
motif 28: Trl
score:0.59
T: 9.2%
P:1e−7
motif 17: Dfd
C2
score:0.67
T: 13.1%
P:1e−8
motif 31: Blimp-1
score:0.60
T: 4.6%
P:1e−6
motif 6: Mad
score:0.55
T: 9.5%
P:1e−9
motif 35: Dref
score:0.90
T: 6.4%
P:1e−4
motif 18: kay
C1
score:0.75
T: 9.5%
P:1e−8
motif 32: vvl
score:0.58
T: 5.0%
P:1e−6
motif 8: br
score:0.78
T: 8.5%
P:1e−9
motif 25: Mef2
C10
score:0.65
T: 6.4%
P:1e−7
motif 20: zld
score:0.74
T:5.7%
P:1e−8
motif 7: Optix
score:0.63
T: 3.9%
P:1e−9
C, expression cluster (up, down)

Hall_FigS7

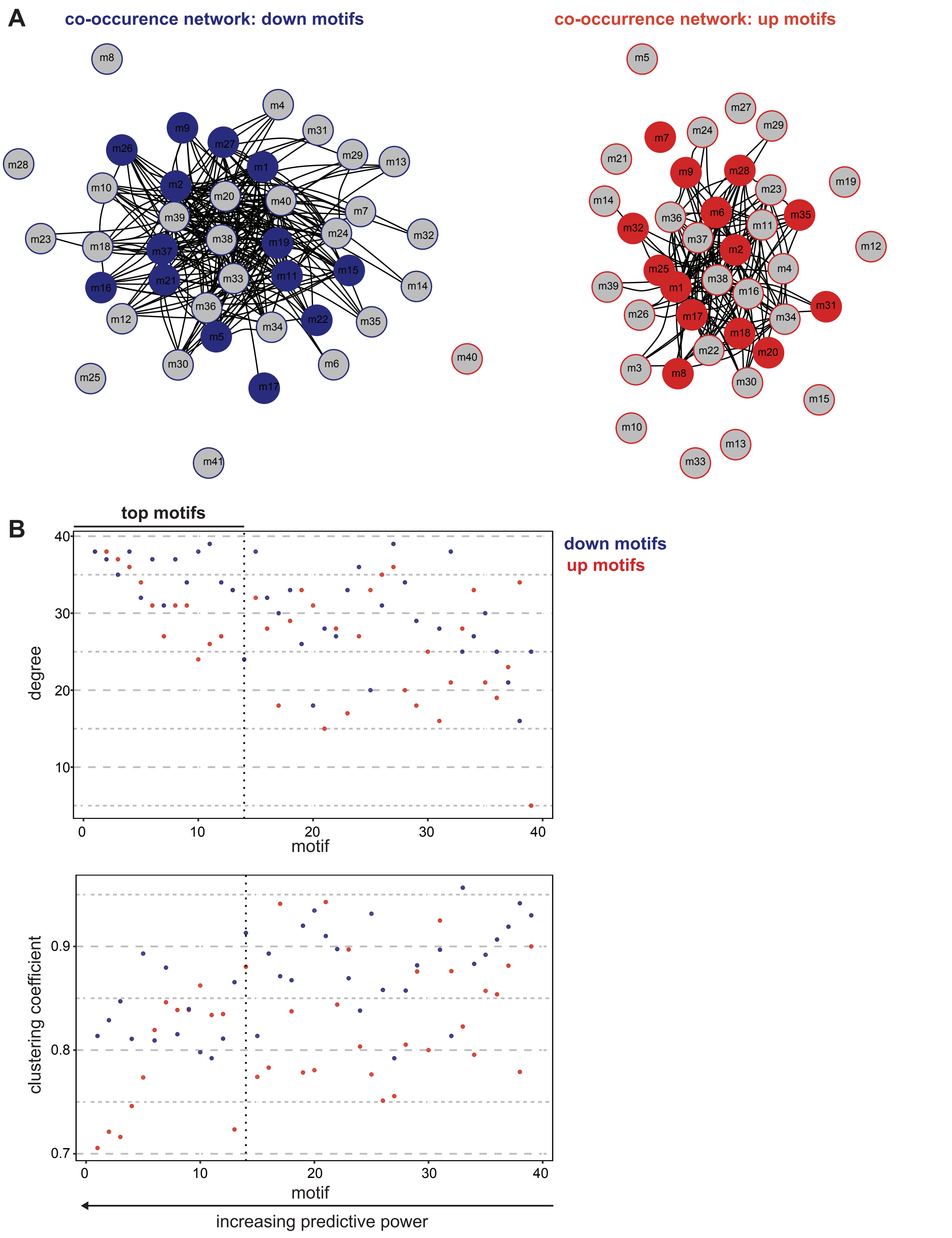

A
co-occurence network: down motifs
m8 m4 m31 m9 m27 m26 m29 m13 m1 m28 m10 m2 m20 m40 m7 m39 m23 m24 m32 m38 m19 m18 m37 m15 m14 m21 m33 m11 m16 m36 m12 m22 m35 m34 m5 m30 m6 m40 m25 m17 m41
co-occurrence network: up motifs
m5 m27 m29 m24 m7 m21 m28 m9 m19 m23 m14 m36 m6 m35 m11 m32 m37 m2 m12 m4 m25 m38 m39 m1 m16 m31 m26 m34 m17 m18 m22 m20 m3 m8 m30 m15 m10 m13 m33
B
top motifs
down motifs
up motifs
degree
40
30
20
10
0
10
20
30
40
motif
clustering coefficient
0.9
0.8
0.7
0
10
20
30
40
motif
increasing predictive power

Hall_FigS8

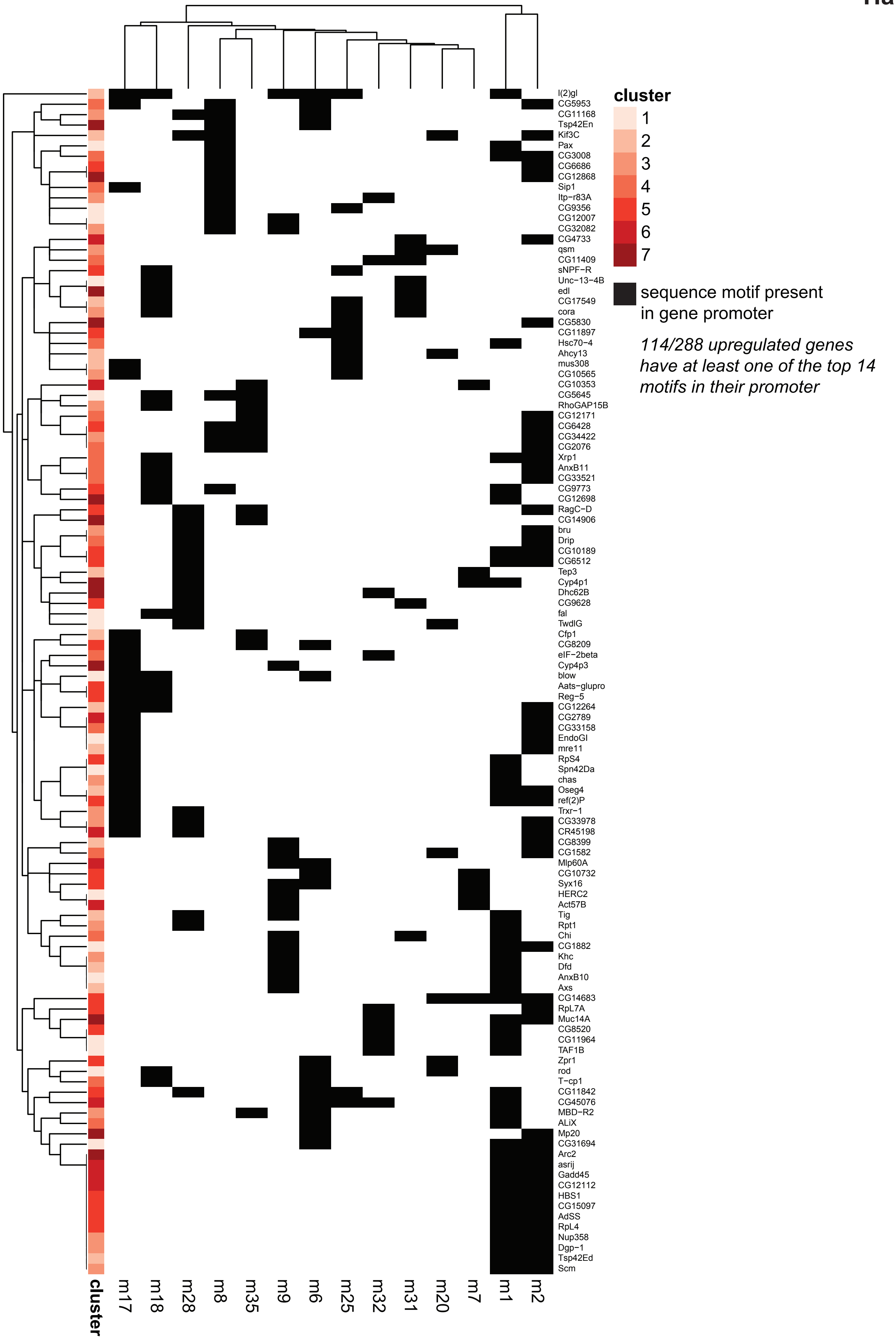
cluster
1
2
3
4
5
6
7
sequence motif present
in gene promoter
114/288 upregulated genes have at least one of the top 14 motifs in their promoter
l(2)gl
CG5953
CG11168
Tsp42En
Kif3C
Pax
CG3008
CG6686
CG12868
Sip1
Itp-r83A
CG9356
CG12007
CG32082
CG4733
qsm
CG11409
sNPF-R
Unc-13-4B
edl
CG17549
cora
CG5830
CG11897
Hsc70-4
Ahcy13
mus308
CG10565
CG10353
CG5645
RhoGAP15B
CG12171
CG6428
CG34422
CG2076
Xrp1
AnxB11
CG33521
CG9773
CG12698
RagC-D
CG14906
bru
Drip
CG10189
CG6512
Tep3
Cyp4p1
Dhc62B
CG9628
fal
TwdlG
Cfp1
CG8209
eIF-2beta
Cyp4p3
blow
Aats-glupro
Reg-5
CG12264
CG2789
CG33158
EndoGI
mre11
RpS4
Spn42Da
chas
Oseg4
ref(2)P
Trxr-1
CG33978
CR45198
CG8399
CG1582
Mlp60A
CG10732
Syx16
HERC2
Act57B
Tig
Rpt1
Chi
CG1882
Khc
Dfd
AnxB10
Axs
CG14683
RpL7A
Muc14A
CG8520
CG11964
TAF1B
Zpr1
rod
T-cp1
CG11842
CG45076
MBD-R2
ALiX
Mp20
CG31694
Arc2
asrij
Gadd45
CG12112
HBS1
CG15097
AdSS
RpL4
Nup358
Dgp-1
Tsp42Ed
Scm
cluster
m17
m18
m28
m8
m35
m9
m6
m25
m32
m31
m20
m7
m1
m2

Hall_FigS9

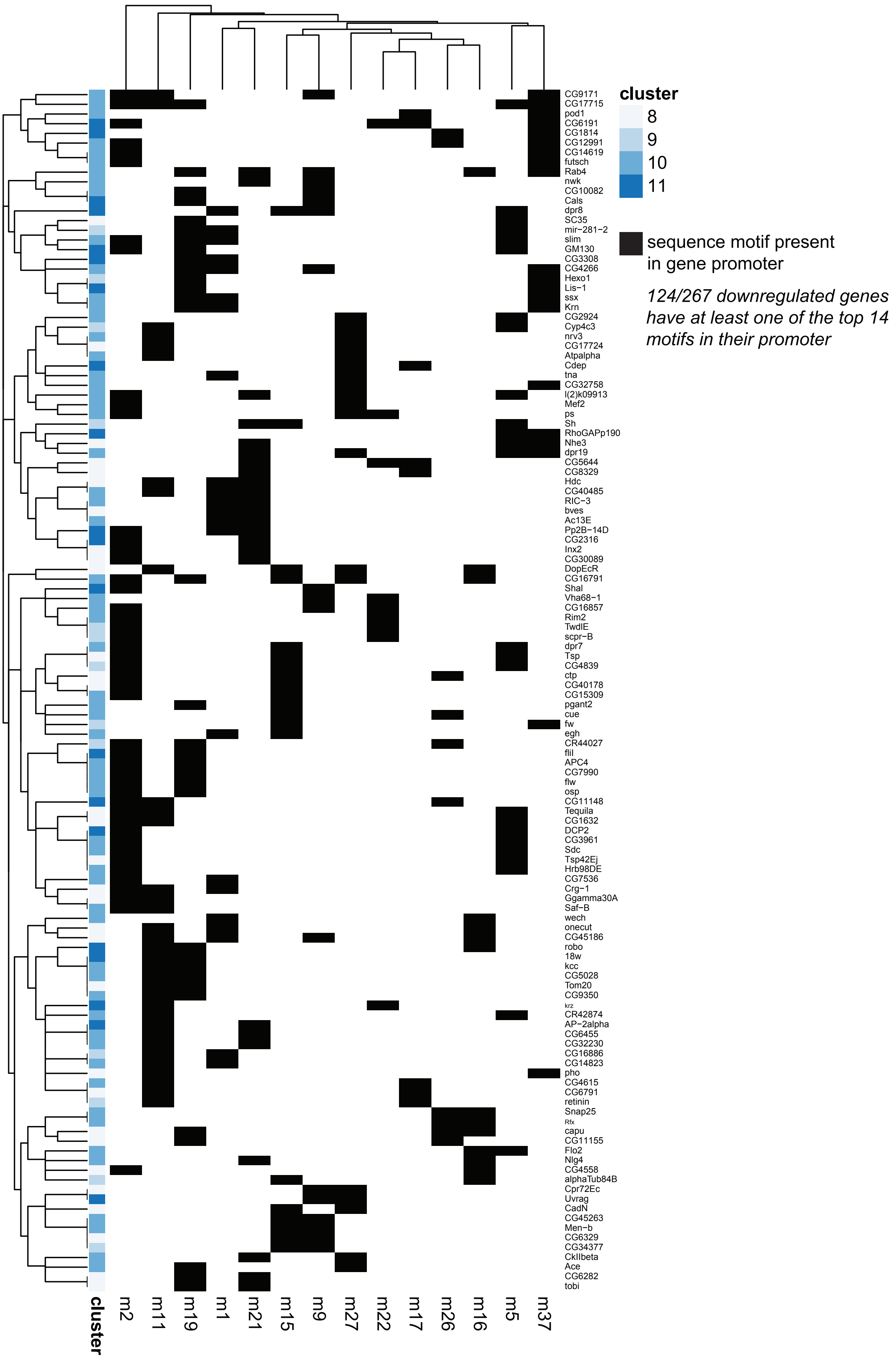
cluster
8
9
10
11
sequence motif present in gene promoter
124/267 downregulated genes have at least one of the top 14 motifs in their promoter
m2 m11 m19 m1 m21 m15 m9 m27 m22 m17 m26 m16 m5 m37
CG9171 CG17715 pod1 CG6191 CG1814 CG12991 CG14619 futsch Rab4 nwk CG10082 Cals dpr8 SC35 mir−281−2 slim GM130 CG3308 CG4266 Hexo1 Lis−1 ssx Krn CG2924 Cyp4c3 nrv3 CG17724 Atpalpha Cdep tna CG32758 l(2)k09913 Mef2 ps Sh RhoGAPp190 Nhe3 dpr19 CG5644 CG8329 Hdc CG40485 RIC−3 bves Ac13E Pp2B−14D CG2316 Inx2 CG30089 DopEcR CG16791 Shal Vha68−1 CG16857 Rim2 TwdlE scpr−B dpr7 Tsp CG4839 ctp CG40178 CG15309 pgant2 cue fw egh CR44027 flil APC4 CG7990 flw osp CG11148 Tequila CG1632 DCP2 CG3961 Sdc Tsp42Ej Hrb98DE CG7536 Crg−1 Ggamma30A Saf−B wech onecut CG45186 robo 18w kcc CG5028 Tom20 CG9350 krz CR42874 AP−2alpha CG6455 CG32230 CG16886 CG14823 pho CG4615 CG6791 retinin Snap25 Rfx capu CG11155 Flo2 Nlg4 CG4558 alphaTub84B Cpr72Ec Uvrag CadN CG45263 Men−b CG6329 CG34377 CkIIbeta Ace CG6282 tobi

Hall_FigS10

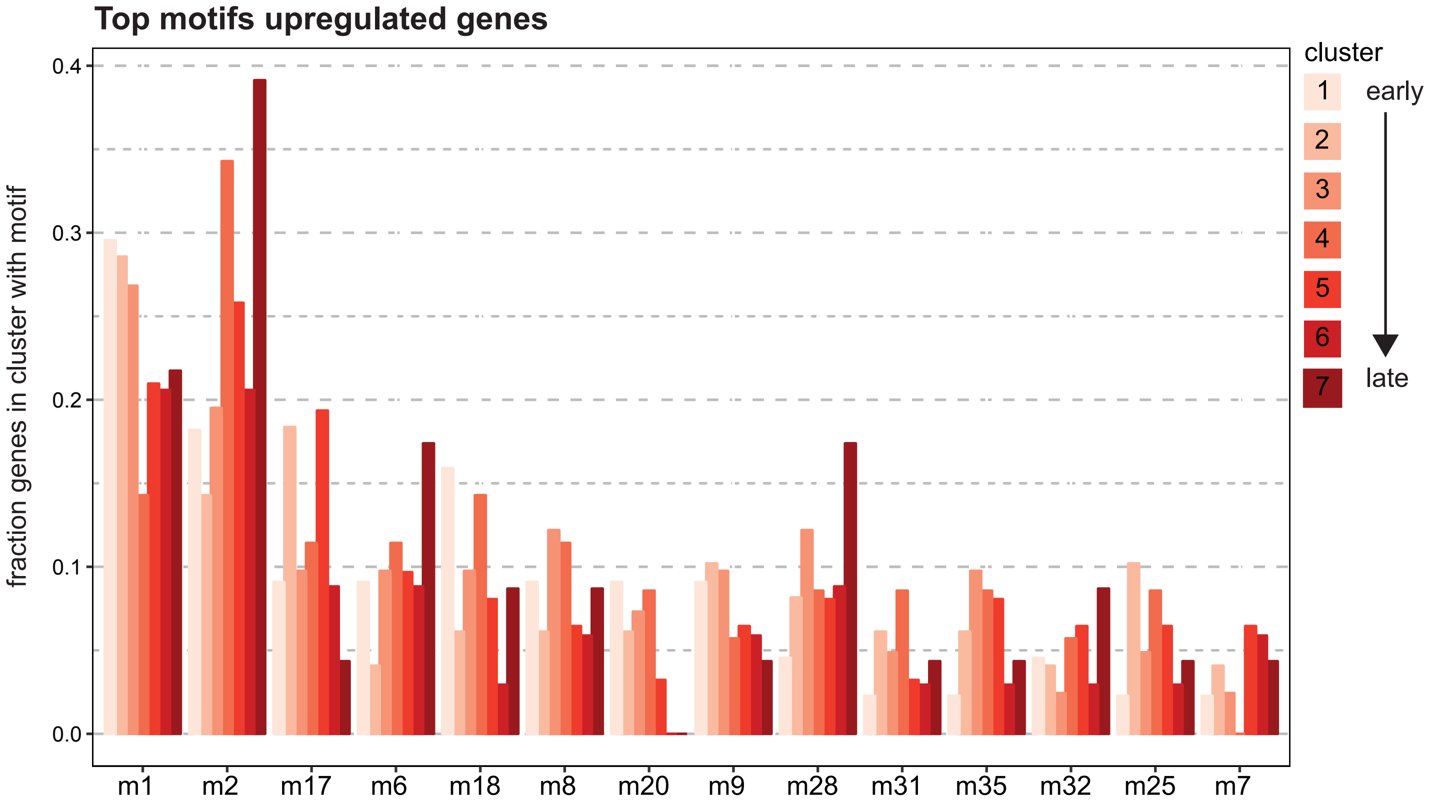
Top motifs upregulated genes
fraction genes in cluster with motif
0.0
0.1
0.2
0.3
0.4
m1
m2
m17
m6
m18
m8
m20
m9
m28
m31
m35
m32
m25
m7
cluster
1
2
3
4
5
6
7
early
late

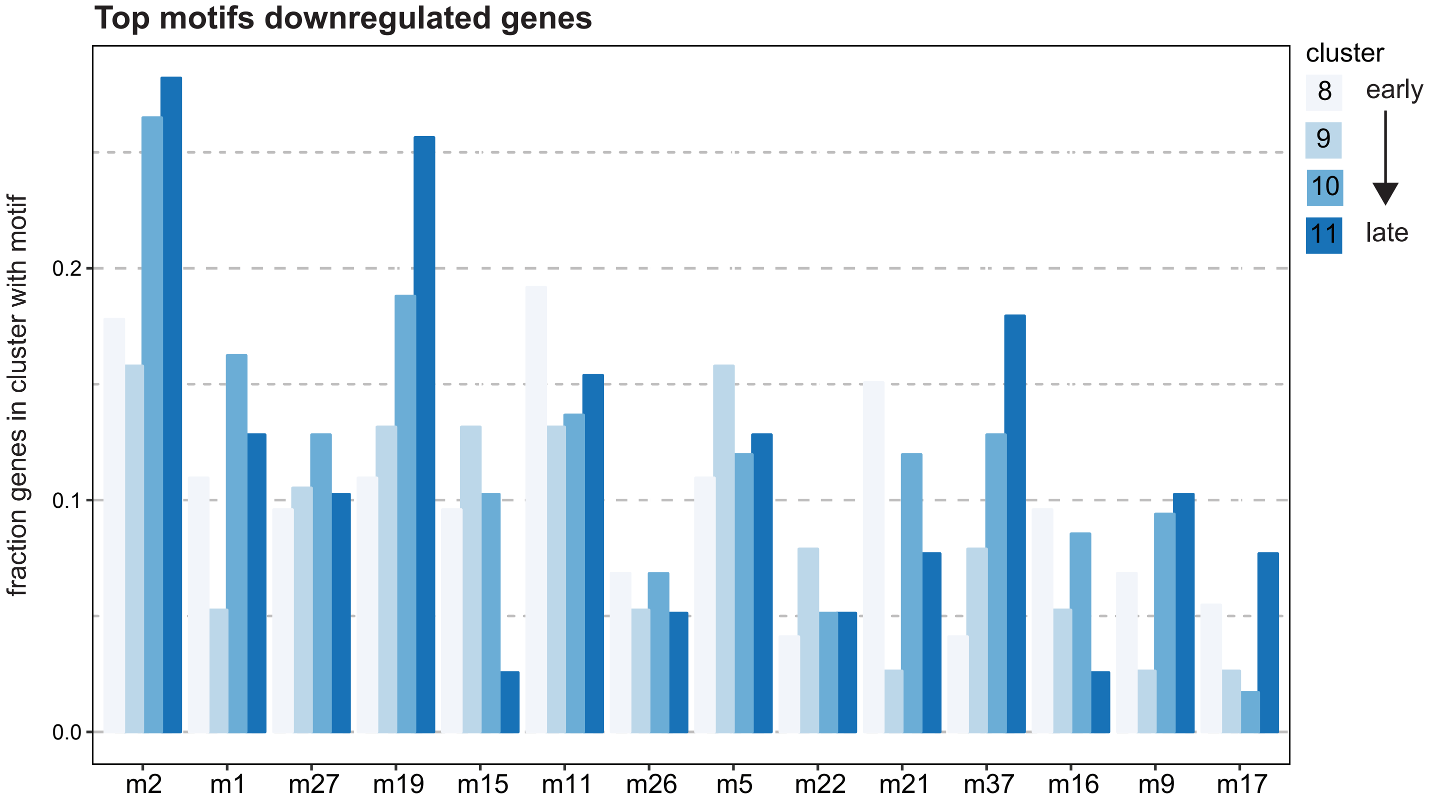
Top motifs downregulated genes
fraction genes in cluster with motif
0.0
0.1
0.2
m2
m1
m27
m19
m15
m11
m26
m5
m22
m21
m37
m16
m9
m17
cluster
8
9
10
11
early
late

Hall_FigS11

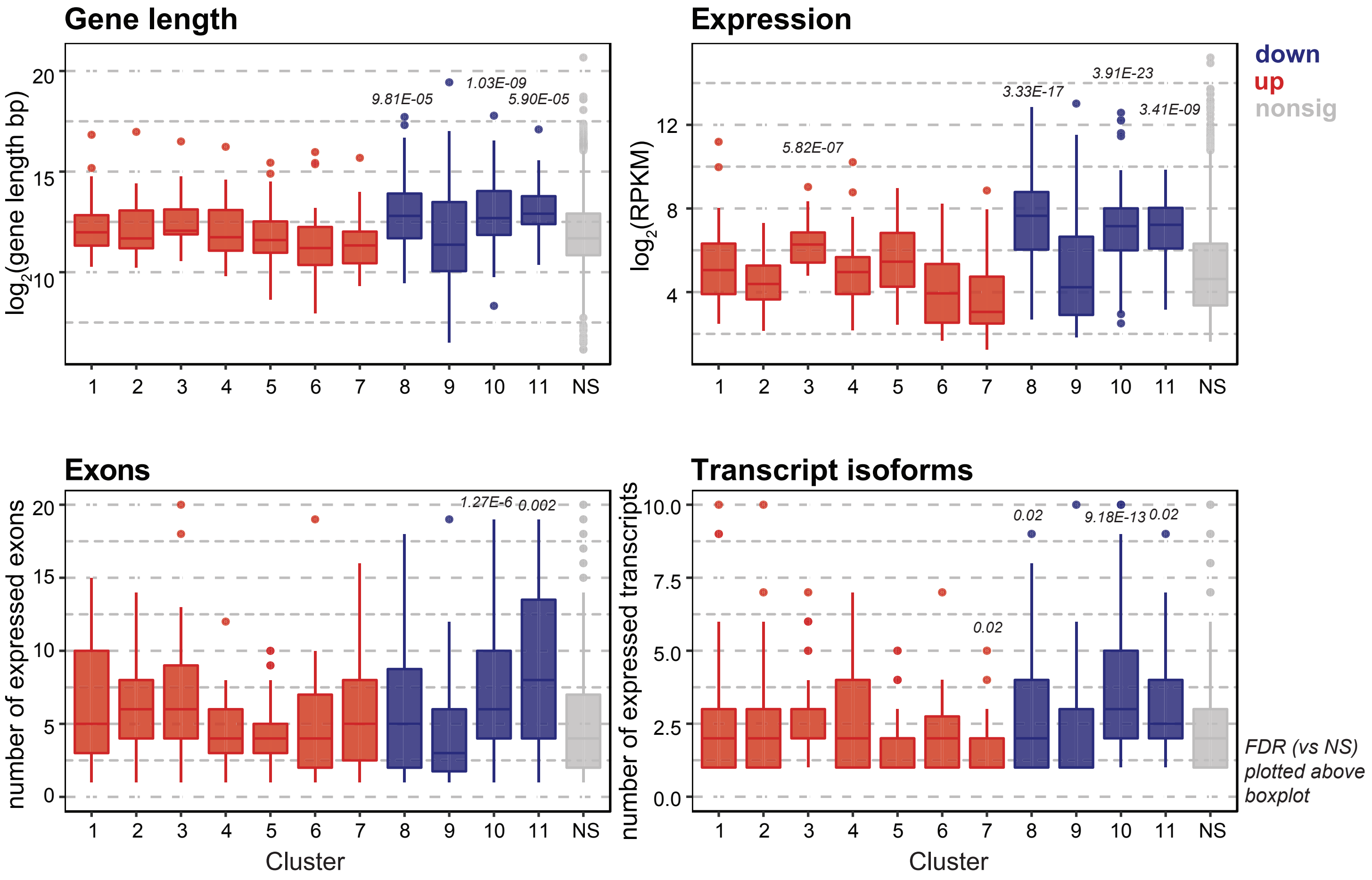
Gene length
log2(gene length bp)
9.81E-05
1.03E-09
5.90E-05
Expression
log2(RPKM)
5.82E-07
3.33E-17
3.91E-23
3.41E-09
down
up
nonsig
Exons
number of expressed exons
1.27E-6
0.002
Cluster
Transcript isoforms
number of expressed transcripts
0.02
0.02
9.18E-13
0.02
Cluster
FDR (vs NS)
plotted above
boxplot

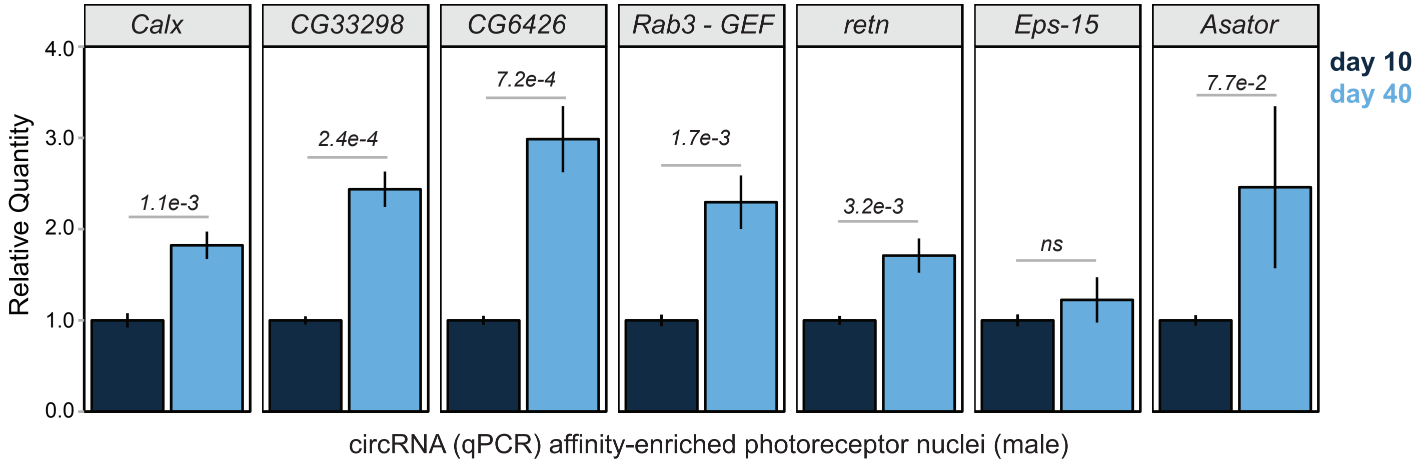
Calx
CG33298
CG6426
Rab3 - GEF
retn
Eps-15
Asator
day 10
day 40
Relative Quantity
4.0
3.0
2.0
1.0
0.0
1.1e-3
2.4e-4
7.2e-4
1.7e-3
3.2e-3
ns
7.7e-2
circRNA (qPCR) affinity-enriched photoreceptor nuclei (male)

Hall_FigS13

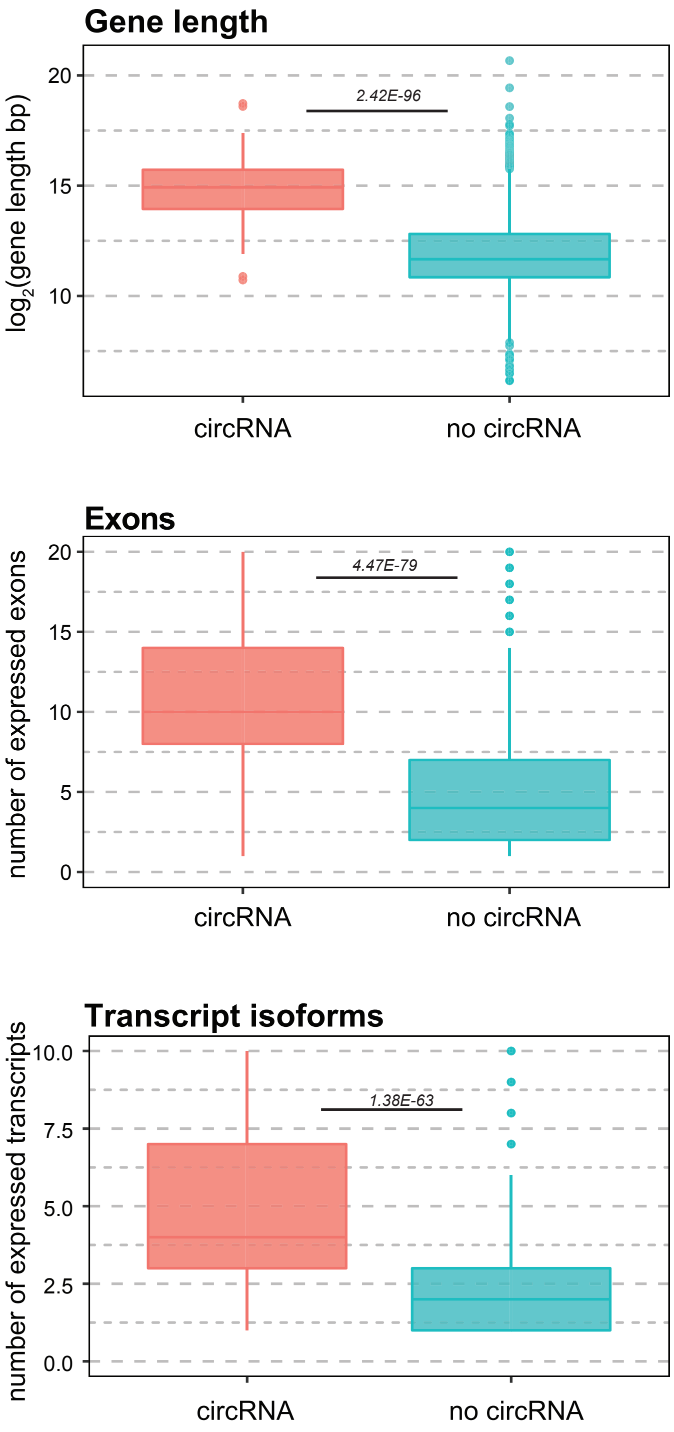

Gene length
log2(gene length bp)
20
15
10
2.42E-96
circRNA
no circRNA
Exons
number of expressed exons
20
15
10
5
0
4.47E-79
circRNA
no circRNA
Transcript isoforms
number of expressed transcripts
10.0
7.5
5.0
2.5
0.0
1.38E-63
circRNA
no circRNA
